# Chromosome-level genome assemblies for the latent pine pathogen, *Diplodia sapinea*, reveal two accessory chromosomes with distinct genomic features and evolutionary dynamics

**DOI:** 10.1093/g3journal/jkaf239

**Published:** 2025-10-07

**Authors:** Preston L Shaw, Bernard Slippers, Brenda D Wingfield, Benoit Laurent, Benjamin Penaud, Michael J Wingfield, Pedro W Crous, Wubetu Bihon, Tuan A Duong

**Affiliations:** Department of Biochemistry, Genetics and Microbiology, Forestry and Agricultural Biotechnology Institute (FABI), University of Pretoria, Private Bag X20, Pretoria 0028, South Africa; Department of Biochemistry, Genetics and Microbiology, Forestry and Agricultural Biotechnology Institute (FABI), University of Pretoria, Private Bag X20, Pretoria 0028, South Africa; Department of Biochemistry, Genetics and Microbiology, Forestry and Agricultural Biotechnology Institute (FABI), University of Pretoria, Private Bag X20, Pretoria 0028, South Africa; BioGeCo, French National Institute of Agronomy, University of Bordeaux, Cestas 33610, France; BioGeCo, French National Institute of Agronomy, University of Bordeaux, Cestas 33610, France; Department of Biochemistry, Genetics and Microbiology, Forestry and Agricultural Biotechnology Institute (FABI), University of Pretoria, Private Bag X20, Pretoria 0028, South Africa; Evolutionary Phytopathology, Westerdijk Fungal Biodiversity Institute, Utrecht 3584 CT, The Netherlands; Department of Biochemistry, Genetics and Microbiology, Forestry and Agricultural Biotechnology Institute (FABI), University of Pretoria, Private Bag X20, Pretoria 0028, South Africa; World Vegetable Center, Eastern and Southern Africa, ILRI Campus, PO. Box 5689, Addis Ababa, Ethiopia; Department of Biochemistry, Genetics and Microbiology, Forestry and Agricultural Biotechnology Institute (FABI), University of Pretoria, Private Bag X20, Pretoria 0028, South Africa

**Keywords:** *Botryosphaeriaceae*, dispensable chromosome, latent pathogen, virulence, genome assembly

## Abstract

*Diplodia sapinea* (*Dothideomycetes*) is a latent fungal pathogen with a global distribution that predominantly infects *Pinus* species. The impact of the fungus is increasing due to climate-driven range expansion and thus wide-scale disease outbreaks are occurring. With the aim of developing high-quality genome resources, we generated chromosome-level genome assemblies for 3 *D. sapinea* isolates and low-coverage Illumina genome data for 6 additional isolates. By comparing these genome assemblies, we identified 14 core chromosomes and 2 accessory chromosomes (ACs) in the pathogen. These 2 ACs encode 80 and 147 proteins, respectively, while 11,374 to 11,601 genes were identified in the core chromosomes. Both ACs had lower gene density and higher proportions of transposable elements compared to the core chromosomes. Sequence analysis indicated that genes on the ACs displayed more sequence variation compared to those on the core chromosomes, suggesting they serve as evolutionary hotspots in the species. Sequence homology analyses suggested that the ACs were possibly acquired horizontally, probably from a species in the *Dothideomycetes*. We designed PCR-based assays to detect the presence of ACs and applied these on a set of 37 isolates from 14 countries. One of the ACs was detected in 33 isolates from 13 countries, while the other AC was absent in all isolates tested. Pathogenicity trials on *Pinus patula* seedlings showed no correlation between the presence of ACs and isolate aggressiveness. The high-quality genomes provided here offer important resources for future research on this globally important pathogen, including the biological roles of the ACs.

## Introduction

*Diplodia sapinea* (synonyms: *Diplodia pinea*; *Sphaeropsis sapinea*) is an opportunistic ascomycete fungal pathogen that predominantly infects *Pinus* spp. (Luchi et al. 2014). The pathogen has a global distribution (Wingfield et al. 2025) and is thought to have spread to areas where nonnative *Pinus* spp. are planted through the trade of infected pine germplasm (Wingfield et al. 2001; Burgess and Wingfield 2002). In recent years, damage caused by *D. sapinea* has increased in areas where it was previously not known to occur, such as northern parts of Europe, likely due to climate change (Brodde et al. 2019; Müller et al. 2019). Understanding the mechanisms by which this pathogen causes disease is crucial for managing its increasing impact.

A key aspect of *D. sapinea*'s biology is its ability to persist in asymptomatic trees for extended periods of time, potentially indefinitely, prior to disease development (Stanosz et al. 2001; Bihon et al. 2011). Disease symptoms develop in trees that become physiologically stressed or damaged due to damage from hail, drought stress, or other unfavorable environmental conditions, as well as biotic disturbances (Palmer et al. 1987; Zwolinski et al. 1990; Stanosz et al. 2001). Symptoms associated with *D. sapinea* infection include shoot dieback (progressive death of young branches), stem cankers (sunken necrotic lesions), resinosis (excessive resin production), blue staining of the sapwood, and root disease, and in severe cases, these can result in host mortality (Chou 1976; Wingfield and Knox-Davies 1980; Swart et al. 1987). Severe disease outbreaks can cause extensive damage to plantations of *Pinus* spp. by killing new shoots, causing deformation and decreasing wood quality (Zwolinski et al. 1990; Swart and Wingfield 1991; Brodde et al. 2019; Caballol et al. 2022).

There are currently 17 genome assemblies available for *D. sapinea* in the NCBI genome database (Bihon et al. 2014; van der Nest et al. 2014; Yu et al. 2022; Wang et al. 2025). The most contiguous of these assemblies contained 14 contigs with an N50 of 2,972,533 bp (Wang et al. 2025). Eleven assemblies are moderately contiguous, ranging from 22 to 33 contigs with N50s ranging from 1.508 to 1.919 Mb. The 5 remaining assemblies are highly fragmented, ranging from 931 to 5,957 scaffolds, with N50 values ranging from 39.4 to 131.4 Kb. The availability of these genome resources has enabled studies on the biology and genetics of the species. The first of these studies characterized the mating type genes and was used in the development of mating type diagnostic markers (Bihon et al. 2014). By comparing the genome assemblies of 2 isolates, the authors showed that *D. sapinea* is heterothallic, characterized by the presence of either *MAT1-1* or *MAT1-2* idiomorph in each of the isolates, and the novel *MAT1-2-5* gene was found. Bihon et al. (2014) also developed mating type markers and used these to examine the mating type distribution in natural populations of the fungus, suggesting the occurrence of a cryptic sexual cycle in *D. sapinea*. The genome resources of *D. sapinea* have also been used to develop a transformation protocol allowing for reverse genetic studies (Oostlander et al. 2024).

Many species of fungi have compartmentalized genomes consisting of core and accessory components (Covert 1998; Galazka and Freitag 2014). The accessory genome includes accessory regions on core chromosomes (CCs) and/or accessory chromosomes (ACs). Accessory chromosomes, also referred to as “B” chromosomes or supernumerary chromosomes, are additional chromosomes that are not present in all individuals of a given species (Bertazzoni et al. 2018). Accessory chromosomes are thought to arise via horizontal chromosome transfer and/or vertically through duplication events of the core genome or a combination of both processes (Soyer et al. 2018). This form of genome compartmentalization provides the advantage that essential processes can be maintained by the core genome while the accessory genome can rapidly evolve and potentially acquire new beneficial functions (Croll and McDonald 2012). This is thought to be particularly true and of relevance in plant pathogen evolution and where the growing availability of *Botryosphaeriaceae* genomes has significantly advanced insights into their biology and host–pathogen interactions (Nagel et al. 2021a, b; Belair et al. 2023; Yu et al. 2022). For instance, comparative genomics among species of the *Botryosphaeriaceae* and other *Dothideomycetes* have also revealed an abundance of secreted hydrolytic enzymes and secondary metabolite BGCs, showing similarity to genomic profiles of other necrotrophic plant pathogens (Nagel et al. 2021a; Yu et al. 2022). Interestingly, while *Diplodia* species, including *D. sapinea*, had the lowest numbers of pathogenicity- and virulence-related genes, prior studies noted that *Botryosphaeriaceae* genomes are not compartmentalized in terms of genome evolution, as is seen in some other plant pathogens (Nagel et al. 2021b). None of these *Botryosphaeriaceae* genomes, however, were assembled to chromosome level, and the potential influence of ACs, which are known to play an important part in plant pathogen evolution (Witte et al. 2021), could not be considered.

Understanding the possible presence of ACs and their role in the biology of *D. sapinea* is essential, given their known role in fungal pathogen evolution and the increasing impact of this pathogen on pine plantations worldwide. In this study, we generated 3 chromosome-level genome assemblies of *D. sapinea* and used these as a basis to identify core and potential accessory chromosomes. The composition, structure, and evolution of the chromosomes were investigated. We also developed molecular markers to characterize the presence of the identified ACs and considered their possible role in the pathogenicity of the species.

## Materials and methods

### Cultures

Isolates of *D. sapinea* used in this study (Supplementary Table 1) were sourced from the culture collection (CMW) of the Forestry and Agricultural Biotechnology Institute (FABI) at the University of Pretoria, South Africa and the CBS-KNAW Fungal Biodiversity Centre, Westerdijk Fungal Biodiversity Institute, Netherlands. Single hyphal tip cultures were made for all the isolates and resulting cultures were maintained on MEA at 25 °C.

### Nanopore and Illumina genome sequencing

Three isolates of *D. sapinea* (CMW190, CMW39103, CMW45410) were selected for Oxford Nanopore long-read sequencing on the MinION device. These isolates were grown in YM broth (2% malt extract, 0.5% yeast extract) for 3 to 5 d until sufficient mycelial mass was obtained. The mycelium was freeze-dried and ground into a fine powder in liquid nitrogen. DNA was extracted from ground mycelium using QIAGEN Genomic-tips (QIAGEN, Hilden, Germany). The cells were lysed for 4 h at 42 °C in cell lysis buffer (10 mM Tris-HCl pH 7.9; 20 mM EDTA, 1% Triton X-100, 500 mM Guanidine-HCl, 200 mM NaCl) supplemented with 0.5 mg/ml cell lysing enzyme from *Trichoderma harzianum* (Sigma-Aldrich, Missouri, United State), 0.8 mg/ml proteinase K (Sigma), and 20 ug/ml RNase A (QIAGEN). The DNA was then purified from the cell lysate using the QIAGEN G-20 Genomic-tip following the manufacturer's suggested protocols.

For long-read sequencing, a genomic library was prepared for each isolate using the Genomic DNA by Ligation (SQK-LSK109) library preparation kit (Oxford Nanopore Technologies, Oxford, United Kingdom) and sequenced on an R9.4.1 flow cell using the MinION sequencing platform (Oxford Nanopore Technologies). Base calling was performed with Guppy v2.7.3 (https://community.nanoporetech.com) using the flip-flop model. Illumina sequencing was conducted on the Illumina HiSeq platform. A TruSeq PCR-free library was constructed for each isolate and sequenced to obtain 151 bp paired-end reads.

### Genome assembly and identification of accessory chromosomes

Nanopore data were trimmed using Porechop v0.2.4 (https://github.com/rrwick/Porechop) to remove any remaining sequencing adapters. Two draft genomes were generated for each isolate using Canu v.2.0 (Koren et al. 2017) and Flye v2.3.1 (Kolmogorov et al. 2019). The draft genome assemblies for each isolate were then aligned to one another using MUMmer v3.23 (Kurtz et al. 2004) to confirm collinearity and identify any conflicts (ie large structural variations) between them. Identified conflicts were resolved by aligning the Nanopore data to both draft assemblies using Burrows–Wheeler Aligner (BWA) v0.7.17-r1188 (Li and Durbin 2010) and manually curating the regions with inconsistencies between the assemblies. The alignment files were converted to BAM format using samtools v1.13 (Li et al. 2009) and viewed using Integrative Genomics Viewer (IGV) v2.8 (Robinson et al. 2011). A final gap-filling step of the curated genome assemblies was done using PBJelly 15.8.24 (English et al. 2012).

The final curated long-read assembly for each isolate was polished using Illumina sequence data. Quality and adapter trimming of the Illumina sequence data was done using Trimmomatic v0.33 (Bolger et al. 2014). Each assembly was initially polished with 3 iterations using Pilon v1.23 (Walker et al. 2014), followed by a final round of polishing using Racon v1.4.12 (Vaser et al. 2017).

The completeness of the final assemblies was determined with BUSCO v4.0.5 (Simão et al. 2015) using the dothideomycetes_odb10 lineage dataset. To determine whether any of the assembled contigs represented complete chromosomes, the sequences were scanned for the telomeric repeat motif (CCCTAA) using Bowtie v1.3.1 (Langmead et al. 2009). Only sequences containing at least 10 consecutive repeat motifs were classified as a telomeric region. Accessory chromosomes were identified by aligning each complete genome assembly to each other using MUMmer v3.23 (Kurtz et al. 2004). Their presence was further confirmed by mapping short reads from isolates lacking the ACs onto the reference genome that contained them. The mapped reads were then inspected in IGV (Robinson et al. 2011) to assess the presence or absence of coverage over the AC regions.

### Genome annotation

Soft-masking of the genomes was performed using RepeatMasker v4.0.7 with a repeat library identified with the REPET package (Flutre et al. 2011). Annotation of protein-coding genes was generated with BRAKER v2.1.5 (Hoff et al. 2019) using the fungi_odb10 protein database with additional proteins from *Macrophomina phaseolina*, *Diplodia corticola*, *Diplodia seriata*, and *Neofusicoccum parvum* downloaded from GenBank as evidence for gene prediction. Initial protein hints were generated using ProtHint v2.5.0 (https://github.com/gatech-genemark/ProtHint) prior to running BRAKER. Functional annotation of the predicted proteins was done using the Blast2GO (Conesa et al. 2005) plugin as part of the CLC Genomics Workbench program v22.0.2. Proteins were queried against the NCBI nonredundant (NR) protein database using BLASTp within the Blast2GO plugin using default parameters. For each query sequence, Blast2GO retrieved the best 20 hits and mapped their associated Gene Ontology (GO) terms to the query sequence using a curated GO database. Additionally, functional domains and their associated GO terms were also identified using InterProScan and merged with GOs identified using BLAST analysis. The Blast2GO results, encompassing BLAST similarity scores and the taxonomic classification of top hit species from the NCBI NR database, were used for comparing the functions and evolutionary relationships of proteins encoded by the CCs and ACs. To test for functional enrichment between genes on the ACs and the core chromosomes, gene ontology (GO) enrichment analysis was performed with Blast2GO using the Fisher's exact test with default parameters.

### Genomic variant discovery in ACs

To determine the prevalence of the ACs and the genetic variation in AC genomic sequences, low-coverage Illumina sequence data for 6 additional *D. sapinea* isolates from 5 countries, as well as the 3 originally sequenced isolates (Supplementary Table 1), were aligned to the reference assembly of isolate CMW45410 using BWA v0.7.17-r1188 (Li and Durbin 2010). The alignment files were converted to BAM format using samtools v1.12. The alignment files were then processed using freebayes v1.3.8 (Garrison and Marth 2012) to identify SNPs throughout the genome of *D. sapinea*. Bcftools (Danecek et al. 2021) was used to filter identified SNPs for quality control concerning depth (DP < 3) and probability score (GQ > 20). Variants were intersected with annotated genomic features using bedtools v2.26.0 (Quinlan and Hall 2010). The SNP density was then calculated for CDS regions and compared between genes on the ACs, a random subset of 80 genes from core chromosomes and 80 random BUSCO genes.

### Secondary metabolite gene clusters and effector predictions

The generated annotations were used as support for secondary metabolite gene cluster predictions with the fungal version of antiSMASH v7.0.0 (Blin et al. 2023) using a relaxed strictness and the CASSIS feature turned on. Signal peptides were predicted from the putative amino acid sequences using SignalP v5.0 (Almagro Armenteros et al. 2019). Unconventionally secreted peptides were identified using OutCyte (Zhao et al. 2019). The predicted secretome, as identified by both SignalP and OutCyte, was subject to effector prediction using EffectorP-fungi 3.0 (Sperschneider and Dodds 2022).

### Orthology analysis

Protein clustering was done with OrthoFinder v2.5.4 (Emms and Kelly 2019) using protein sequence data from 3 *D. sapinea* isolates (CMW39103, CMW190, and CMW45410) for which whole genome and annotation were generated, as well as using proteomes from additional species of the *Botryosphaeriaceae* for which data were publicly available at the time of this study (Supplementary Table 2). OrthoFinder analyses were run with a range of MCL inflation parameters (from 1.5 to 4.0 using increments of 0.5), and the run that produced the highest number of single-copy orthogroups was selected for further analyses. The orthogroups generated by OrthoFinder were used to identify and compare shared and unique genes on the ACs and core chromosomes.

### Phylogenomic analysis of *Botryosphaeriaceae*

The genomes of all *Botryosphaeriaceae* species used for protein clustering were analyzed with BUSCO using the dothideomycetes_odb10 lineage dataset. Single-copy BUSCO genes that were shared among all 30 species were identified and used to construct a species phylogeny using a coalescence approach. Shared, single-copy BUSCO amino acid sequences were extracted from the BUSCO outputs and aligned using PRANK v.170427 (http://wasabiapp.org/software/prank/) with default parameters. The aligned datasets were trimmed with trimAI v1.5 (Capella-Gutiérrez et al. 2009) with the “-atomated1” option. Maximum Likelihood (ML) trees were inferred for individual datasets using IQ-TREE v1.6.11 (Nguyen et al. 2015) with best fit substitution models computed automatically and 1,000 rapid bootstrap replicates. The resulting ML trees were used to construct a coalescence tree using ASTRAL v5.7.7 (Zhang et al. 2018). The species tree obtained from ASTRAL was optimized for branch length with RaxML v8.2.11 (Stamatakis 2014) using a concatenated dataset derived from aligned and trimmed individual BUSCO datasets. Gene concordance factors (gCF) were computed in IQ-TREE using the individual gene trees.

### Development of diagnostic multiplex PCR assays for the presence of ACs

A total of 7 primer pairs were designed for multiplex PCR assays (Supplementary Table 3). Genes used for primer design to detect AC Chr 15 were chosen based on their sequence conservation in isolate CMW190 as well as their position on the chromosome. This was done by comparing Illumina sequence alignments of the additional *D. sapinea* isolates (Supplementary Table 1) to AC Chr 15 in IGV. As AC Chr 16 was unique to 1 isolate, the method of using conserved sequences was not applicable. Gene candidates for primer design were thus chosen based on whether they formed single-copy orthogroups after protein clustering with OrthoFinder. Three primer pairs were designed to amplify gene regions on AC Chr 15. These regions included a gene encoding a secreted hypothetical protein, a polyketide synthesis gene, and a toxin synthesis gene. Furthermore, 3 primer pairs were designed to amplify gene regions on AC Chr 16, targeting genes encoding a putative C6 finger domain protein, a hypothetical protein, and a NACHT and WD40 domain. The final primer pair was designed to amplify the beta-tubulin gene regions, acting as a positive control. All primers were designed using Primer3Plus v2.4.2 (Untergasser et al. 2012) to have similar annealing temperatures and different amplicon sizes to allow PCR multiplexing (Supplementary Fig. 1).

### PCR amplification of accessory chromosomes

A total of 37 isolates from 14 countries across 6 continents were used in PCR assays to detect the presence of the ACs (Supplementary Table 4). Three isolates (CMW39103, CMW190, and CMW45410) for which chromosome-level genome sequences had been generated were included as controls. DNA samples were extracted from pure cultures actively growing on MEA (2% malt extract, 2% Agar, Biolab, South Africa). Mycelium was transferred to separate 1.5 ml Eppendorf tubes, and DNA was extracted using PrepMan Ultra Sample Preparation Reagent (Thermo Fisher Scientific) using a temperature of 95 °C for a total of 10 min. The mycelium was macerated using a pestle after the first 5 min of heating. The extracted DNA was then diluted 5 times with 10 mM Tris-HCl, pH 8.0, and subsequently used for PCR amplification. A multiplex PCR assay was performed for the detection of each AC separately. Each PCR reaction consisted of 2 µl DNA template, 0.2 µl FastStart Taq DNA polymerase (Roche Applied Science, Mannheim, Germany), 2.5 µl dNTPs (2 mM each), 2.5 µl 10× PCR buffer w/MgCl2, 5 µl GC-Solution, 0.5 µl MgCl2 (25 mM), 0.5 µl of each primer (10 µM), and H_2_O up to the final volume of 25 µl. PCR amplification was carried out with a MyCycler Thermal Cycler from Bio-Rad using cycling conditions as described by de Beer et al. (2014) with an annealing temperature of 55 °C. The final PCR products were stained with GelRed (Biotium, California, United States) and separated using gel electrophoresis in a 2% agarose gel (SeaKem LE Agarose, Lonza Bioscience) at 80 V for 45 min. The PCR amplicon was then visualized under UV light using a Gel Doc EZ Imager (Bio-Rad). The presence/absence of the ACs was identified by the presence/absence of the amplicons from the targeted genes. The identity of each isolate was confirmed by sequencing the positive control amplicon targeting the beta-tubulin gene region and conducting a BLAST analysis against the NCBI nonredundant nucleotide database.

### Pathogenicity trial

A pathogenicity test was conducted on approximately 2-yr-old *Pinus patula* saplings using 6 isolates of *D. sapinea* (Supplementary Table 5). Isolates used in the pathogenicity trial were chosen based on whether they possessed the ACs or not, as indicated by the PCR screening. Isolates used in the pathogenicity trials were freshly prepared on 2% MEA and maintained at room temperature until the day of inoculation. *P. patula* saplings were inoculated and maintained in a greenhouse at 25 °C.

Inoculations were performed by removing the outer bark to expose the cambium of the plants with a 4-mm-diameter cork borer approximately 5 cm below the apical bud. A plug of mycelium-covered media was placed onto the freshly made wound, mycelium side facing the wounded surface, and sealed with parafilm to reduce desiccation and contamination. A total of 7 saplings were inoculated per isolate, and an equal number were inoculated with 2% MEA as a negative control. The plants were then left for a period of 3 wk. The resulting lesions were measured by cutting away the bark with a scalpel and measuring the length of the lesions downward on the stems starting from the base of the inoculation point. Reisolations were made from the lesions for 3 plants per isolate by placing a small piece of stem tissue harvested from the base of the lesion on 2% MEA. Fungi that resembled *D. sapinea* in culture were transferred to fresh growth medium, purified, and identified via PCR amplification and sequencing of the ITS region using the ITS-1F and ITS-4 primers.

## Results

### Chromosome-level assemblies reveal the presence of 2 accessory chromosomes in *D. sapinea*

The long reads generated for 3 isolates of *D. sapinea* (CMW39103, CMW190, and CMW45410) were used to assemble the genomes using 2 different assemblers. In most cases, Canu generated the best initial assembly results but slightly under assembled the genome, whereas Flye performed well but occasionally misjoined contigs (Supplementary Table 6). The Canu assemblies were selected as the base assemblies for the curation process by means of whole genome alignment and long-read mapping for manual verification (Supplementary Fig. 2). The final genome assemblies of 3 *D. sapinea* isolates had 14, 15, and 16 contigs, respectively, with assembled genome sizes ranging from 37.01 to 38.4 Mb (Table 1). The telomeric repeat sequence (CCCTAA) was detected at both ends of 15 contigs in at least 1 of the 3 assembled genomes (Supplementary Fig. 3) indicating that these genomes were likely assembled into complete or close to complete chromosomes. However, no telomeric regions were detected for Chr 16 in CMW45410; we therefore provisionally refer to this contig as a chromosome, pending further structural validation. BUSCO analyses using the dothideomycetes_odb10 dataset (*n* = 3786) indicated a high level of assembly completeness in all 3 assemblies with BUSCO scores higher than 99% (Table 1). Genome annotation resulted in a total of 11,485 protein-coding genes in CMW39103, 11,770 in CMW190, and 11,609 in CMW45410. Of the predicted protein-coding genes, 79 are located on Chr 15 in CMW190, 80 on Chr 15 in CMW45410, and 155 on Chr 16. BUSCO completeness for the genome annotations using the dothideomycetes_odb10 dataset was 98.3%, 98.4%, and 97.8%, respectively. The lower annotation BUSCO scores compared to the genome scores indicated that the gene prediction pipeline was not optimal for predicting all possible genes.

**Table 1.** Assembly and annotation statistics of CMW39103, CMW190, and CMW45410 generated in this study.

	CMW39103	CMW190	CMW45410
Genome size (bp)	37,011,478	38,053,143	38,401,314
GC content (%)	56.66	56.57	56.54
Number of contigs	14	15	16
N50 (bp)	2,852,041	2,851,318	2,860,161
L50	6	6	6
BUSCO (*Dothideomycetes*: n:3786)	99.4%	99.4%	99.3%
Accessory chromosomes	-	Chr 15 (0.48 Mb)	Chr 16 (0.64 Mb); Chr 15 (0.46Mb)
No. protein-coding genes	11,485	11,770	11,601
No. single exon genes	2,333	2,414	2,374
Mean CDS length (b)	1,463	1,456	1,458
Mean exons per gene	3.0	3.0	3.0
Mean exon length (b)	487	487	488
Mean intron length (b)	85	85	87

**Fig. 1.**
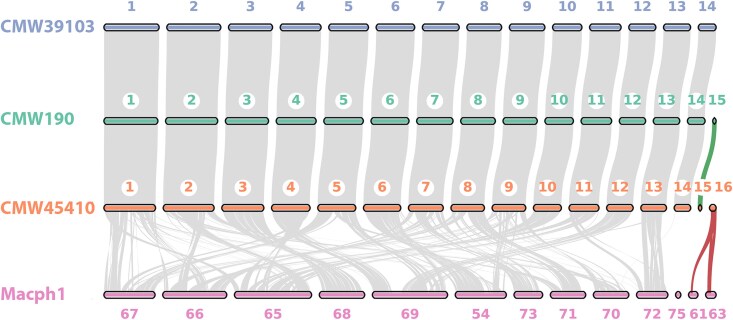
Genome-wide collinearity between 3 assembled *D. sapinea* genomes and assembled genome of *M. phaseolina* (GCA_020875535.1). Colinear blocks were identified and visualized using MCScanX (Wang et al. 2012). Putative chromosome numbers are assigned for *D. sapinea* genomes, and contig numbers in the original *M. phaseolina* genome were used. The green ribbon shows collinearity between Chr 15 in 2 *D. sapinea* isolates; red ribbons show sequence similarity and collinearity between Chr 16 and 2 contigs of *M. phaseolina*.

Synteny comparison between 3 assembled genomes revealed a high level of collinearity, with end-to-end alignment across 14 predicted core chromosomes (Fig. 1). This analysis also identified 2 possible accessory chromosomes, characterized by their variable presence among isolates, Chr 15 and Chr 16. Predicted chromosome (Chr 15) was shared by CMW190 and CMW45410, while the other (Chr 16) was unique to CMW45410 (Fig. 1). Isolate CMW39103 had neither of these chromosomes.

### Genes on the ACs display high sequence homology to *M. phaseolina*, a distantly related species of *Botryosphaeriaceae*

**Fig. 2.**
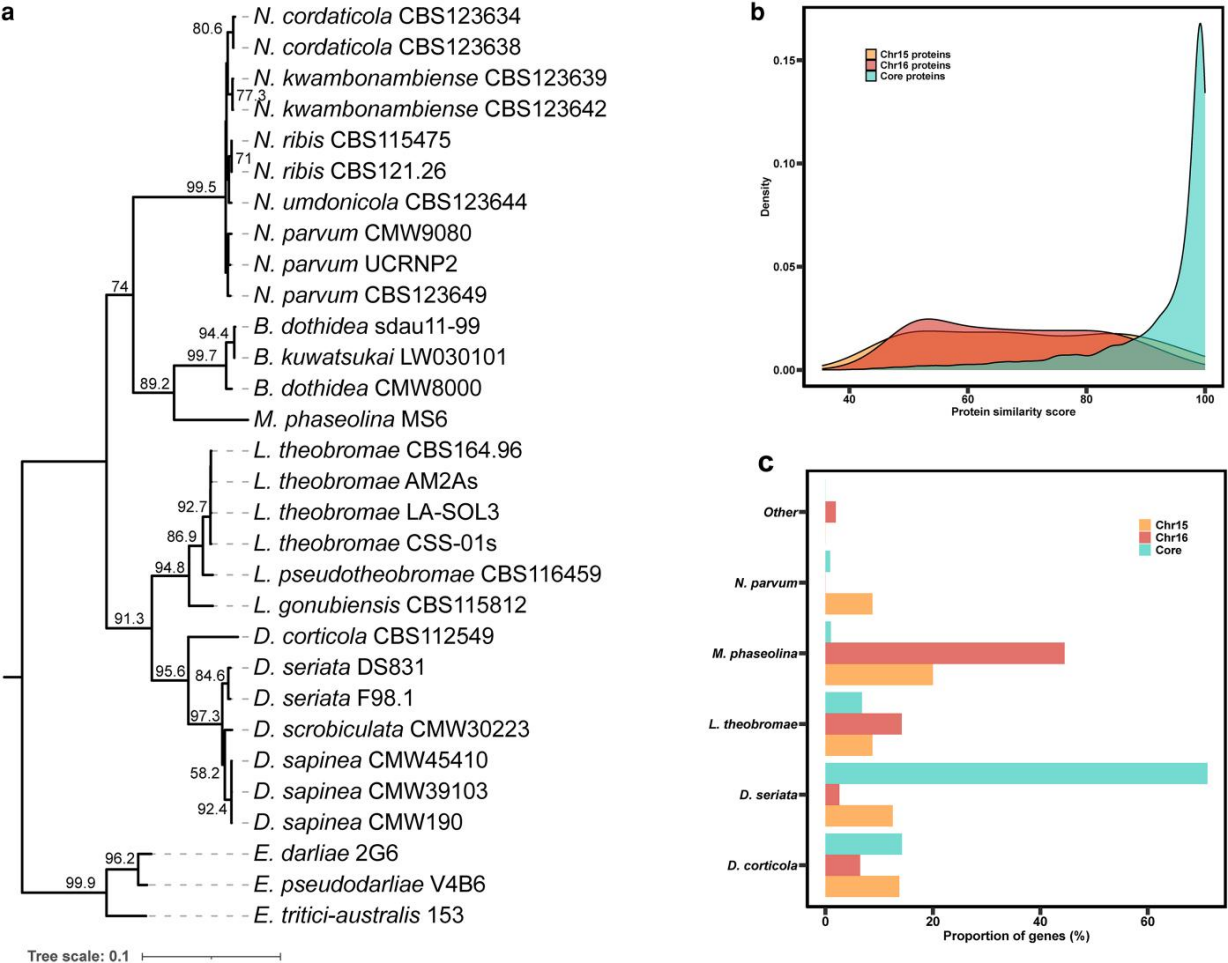
a) Phylogenomic tree based on shared single-copy orthologs determined by BUSCO of 30 *Botryosphaeriaceae* isolates. Gene and site concordance factors (gCF/sCF) as determined by IQ-TREE 2 are presented on the branches of the tree. b) Distribution of BLAST similarity scores against NR protein database for putative amino acid sequences encoded by genes of each AC compared to those of the core chromosomes. c) Proportion of proteins attributed to the top 5 species BLAST hits for putative proteins encoded by genes on each AC and the core genome.

Sequence homology analysis indicated that most of the proteins encoded by genes of the core chromosomes (CCs) showed homology to existing sequences in the NCBI nonredundant (NR) protein database, with the majority displaying above 90% sequence similarity. These CC-encoded proteins were most similar to other *Diplodia* sp., particularly *D. seriata*, which accounted for over 75% of the top 5 species BLAST hits, followed by *D. corticola* with over 15% (Fig. 2c). This finding aligns with close evolutionary relationship between *D. sapinea*, *D. seriata*, and *D. corticola* (Fig. 2a). We note that phylogenomic analysis indicates that *Diplodia scrobiculata* is more closely related to *D. sapinea* than *D. corticola* and *D. seriata*, but that its amino acid sequences are not available in the public NR database and thus were not represented in the BLAST results.

In contrast, proteins encoded by genes on the ACs displayed much lower sequence similarity to those on the NR protein database, averaging 68.4% (Fig. 2b). Most of the AC-encoded proteins exhibited the highest sequence similarity to *M. phaseolina*, a more distantly related species within the *Botryosphaeriaceae* family. Specifically, BLAST hits to sequences of *M. phaseolina* accounted for over 31% of the top 5 species BLAST hits for genes on Chr 15 and over 63% of the hits for genes on Chr 16. The next most similar species based on BLAST hits were *D. corticola* (21% of BLAST hits) and *D. seriata* (19% of BLAST hits) for genes on Chr 15 and *Lasiodiplodia theobromae* (20% of BLAST hits) and *D. corticola* (9% of BLAST hits) for genes on Chr 16.

### The ACs display evolutionary plasticity and are enriched in effectors

**Fig. 3.**
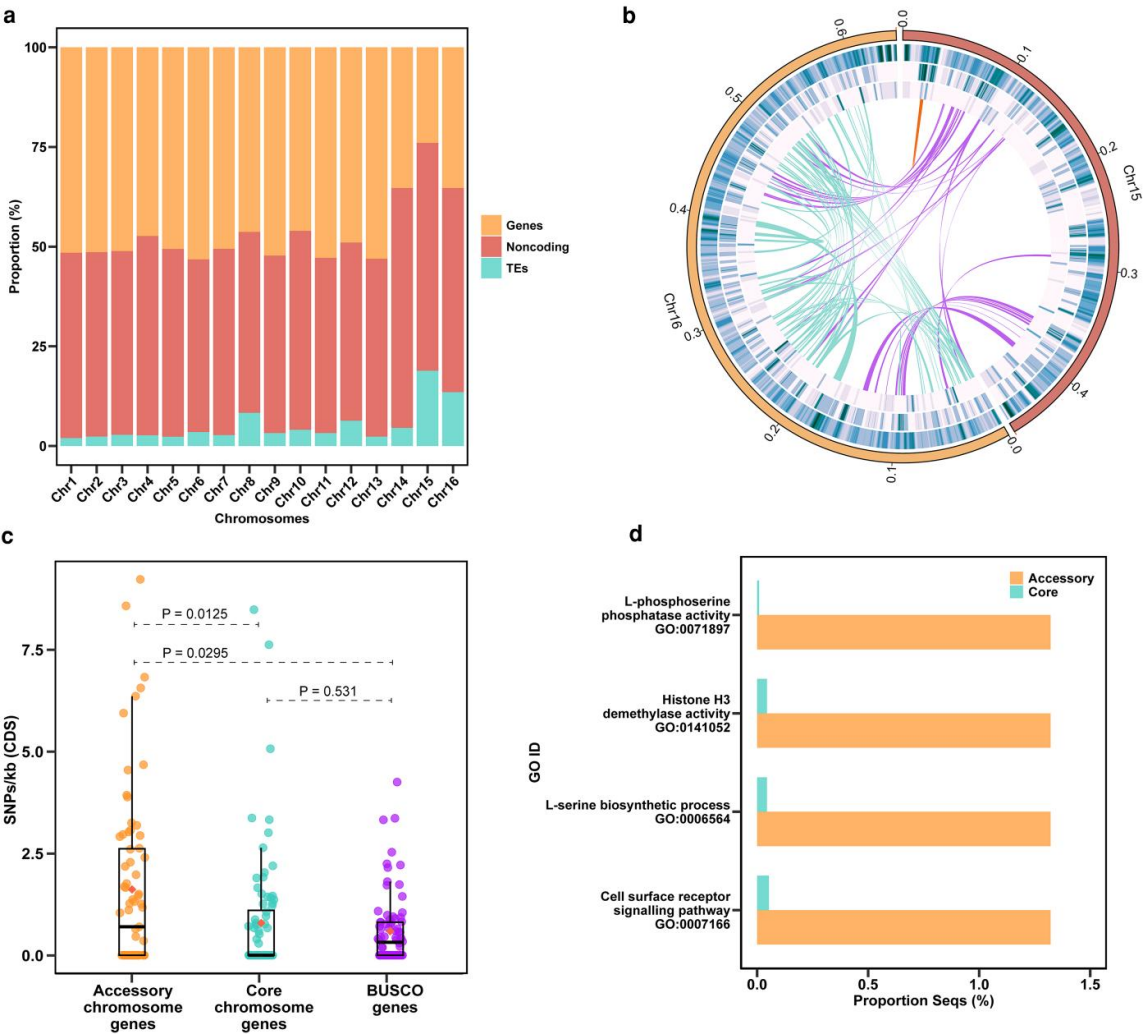
a) Genomic composition (genes, noncoding and transposable elements (TEs) of the 16 predicted chromosomes in *D. sapinea* isolate CMW45410. The ACs are labeled as Chr 15 and Chr 16. b) Circular visualization of the ACs in isolate CMW45410 ACs. Tracks from outer to inner: 1, accessory chromosomes and their lengths in Mb; 2, GC density; 3, gene density; 4, TE density. Links show genes encoding homologous proteins between and within ACs as identified by OrthoFinder (purple, between Chr 15 and Chr 16; orange, within Chr 15; light blue, within Chr 16). c) Number of SNPs per Kb of CDS for genes on Chr 15, a random subset of 80 genes from core chromosomes and 80 random BUSCO genes. Statistical significance was calculated using the Wilcoxon rank-sum test. d) GO enrichment analysis of genes on the accessory chromosomes vs genes on the core chromosomes.

The genomic composition (genes, transposable elements, noncoding) of the ACs was distinct from that of the core chromosomes (Fig. 3a). Both ACs had a higher abundance of TEs and lower gene densities when compared to that of the core chromosomes. Approximately 24% of Chr 15 and 33.5% of Chr 16 were composed of predicted gene regions (exons and introns), while transposable elements constituted approximately 18.9% and 13.5% of these chromosomes, respectively. In contrast, all CCs consisted of at least 46% gene regions, with the exception of Chr 14, which was 35.3%. Among the CCs, Chr 8 displayed the highest proportion of TEs, accounting for 8.3% of its composition.

Sequence comparison indicated that genes on AC Chr 15 had a significantly lower level of sequence conservation when compared to CC genes (Fig. 3c). Additionally, 86% of the identified SNPs in genes on the AC were Repeat-Induced Point (RIP)-like mutations (C → T/G → A), a fungal genome defense mechanism that targets and mutates repetitive DNA sequences. This is more than double compared to the RIP-like mutations identified in genes on the core chromosomes (Supplementary Fig. 4). Sequence variation analysis could not be conducted for Chr 16 due to it being present in only a single isolate.

Protein clustering analysis of proteins encoded by the ACs revealed that 73 putative proteins encoded by Chr 16 share homology among themselves, indicating that this chromosome has undergone segmental duplications (Fig. 3b, Supplementary Table 7). In contrast, only 4 putative proteins of Chr 15 shared homology with one another. When comparing between the ACs, 19 putative proteins share homology with 29 proteins of Chr 16.

A total of 1,147 secreted proteins were identified in isolate CMW45410, 8 of which originated from Chr 15 and 4 from Chr 16. A further 12 unconventionally secreted proteins were identified for Chr 15 and 29 for Chr 16. Of the predicted secreted peptides, 11 were predicted as effectors for Chr 15 and 18 were predicted as effectors for Chr 16. The ACs had a higher prevalence of putative effectors, with 13.75% of genes on Chr 15 and 11.84% of genes on Chr 16 encoding putative effector proteins, compared to 8.92% of genes on the CCs encoding putative effector proteins. A fungal-RiP-like gene cluster was also predicted on Chr 16, and no biosynthetic gene cluster was detected on Chr 15 despite the presence of a polyketide synthase gene.

Gene ontology enrichment analysis revealed a distinct enrichment of specific GO terms on the ACs compared to the CCs (Fig. 3d). Enriched terms include histone H3 demethylase activity (GO:0141052), cell surface receptor signaling pathway (GO:0007166), L-serine biosynthetic process (GO:0006564), and L-phosphoserine phosphatase activity (GO:0036424).

### Chr 15 was widespread whereas Chr 16 was detected in only a single isolate

Low-coverage Illumina sequencing of 6 *D. sapinea* isolates (Supplementary Table 1) showed that Chr 15 occurred commonly and was found in 8 of the 9 isolates for which genome data was generated. These isolates were from South Africa, Sweden, Italy, Chile, and France. One isolate (CMW39103) from South Africa did not contain this chromosome. In contrast, the second AC was found in only a single isolate (CMW45410) originating from Sweden. Multiplex PCR assays targeting 3 genes on each of the ACs on a total of 40 isolates (including CMW190, CMW39103, and CMW45410) resulted in the identification of Chr 15 in 35 isolates. None of the additional isolates investigated harbored Chr 16 (Supplementary Table 4). Whole genome alignment of 15 recently generated *D. sapinea* genomes from various countries—including 2 from Brazil, 2 from Canada, 4 from China, 1 from Estonia, 4 from Russia, and 2 from Singapore—against the genome of CMW45410 revealed that all these assemblies possess AC Chr 15, and none have Chr 16, except the 2 assemblies from Singaporean isolates, which had neither of the ACs (Supplementary Table 8). Isolates from the 21 countries considered for the presence of ACs showed that 20 contained the AC Chr 15. These isolates were from South Africa, Ethiopia, New Zealand, Montenegro, Russia, Serbia, Estonia, Sweden, Switzerland, Canada, the United States, Brazil, Chile, Colombia, China, Netherlands, Great Britain, France, and Italy. Isolates from Indonesia and Singapore did not contain either of the ACs. A table summarizing AC presence for all isolates is available in Supplementary Table 8.

### The presence of ACs showed no correlation with pathogenicity

The *D. sapinea* isolates used in the inoculation trial showed significant variation in their ability to produce stem lesions, but there was no clear correlation between AC presence and lesion length. Regardless of whether ACs were present or absent, most isolates produced clear lesions longer than those of the controls. The only exception was isolate CMW29644 that produced relatively small lesions (Supplementary Fig. 5). The isolates without the ACs (CMW39103, CMW34220, and CMW4889) produced average lesion lengths of 68.71, 67.14, and 57.86 mm, respectively (Supplementary Table 9). The isolates (CMW8754 and CMW34220) having AC Chr 15 produced average lesion lengths of 58.71 and 19 mm, respectively. The isolate (CMW 45410) with both AC Chr 15 and Chr 16 gave rise to lesions having an average length of 62 mm. None of the control inoculated plants had lesions. Reisolations, followed by sequencing of the ITS region, confirmed the presence of *D. sapinea* on the dead trees.

## Discussion

Chromosome-level genome assemblies were generated for 3 isolates of *D. sapinea*, and genomic data were produced for 6 additional isolates. This level of genome completeness, contiguity, and structural accuracy allowed for the discovery of 2 ACs for the first time in this pathogen. Sequence analyses revealed that these ACs have unique genomic compositions compared to the core chromosomes and that they have possibly been acquired horizontally. Pathogenicity assays showed no correlation between the presence of ACs and the length of lesions on inoculated saplings.

The ACs identified in this study are the first to be identified in a species of the *Botryosphaeriaceae*. Comparison of protein sequences encoded by genes of the ACs and CCs with available proteins from the NR database revealed different profiles of taxonomic association. Proteins encoded by CCs showed the highest similarity to proteins in other *Diplodia* species, while proteins encoded by the AC genes showed higher similarity to those from *M. phaseolina*, a distantly related species in the *Botryosphaeriaceae*. This suggests a potential horizontal origin for both ACs discovered in *D. sapinea*. Additionally, the unique genomic composition of the ACs compared to the CC also suggests that they have been acquired by horizontal transfer. Alternatively, these ACs could be common in members of the *Botryosphaeriaceae* and be inherited vertically. If this is true, they will be identified in other members of the family as more complete genomes are generated.

The ACs identified in this study exhibited distinct genomic characteristics compared to the CCs. For example, the ACs displayed a lower gene density, a higher proportion of TEs, and increased SNP variations. Such higher TE content is typically observed for ACs (Williams et al. 2016) and can be attributed to the fact that they do not carry essential genes, thereby minimizing the risk of TE disrupting vital gene functions (Bertazzoni et al. 2018). The presence of TEs can contribute to genome plasticity and generate further variation via RIP mutations (Lorrain et al. 2021). The segmental duplications observed in Chr 16 are likely mediated by TEs. Orthology analysis indicated that half of the proteins on Chr 16 show orthology to each other as a result of these duplications. These duplicated genes can serve as sources for functional diversification (Marques et al. 2008), thereby providing genomic variation that can be acted upon by natural selection.

Accessory chromosomes in plant pathogens are widely considered to be important drivers of genome evolution, influencing phenotypic traits such as pathogenicity, virulence, and host adaptation. For instance, an AC in *Nectria haematococca* confers increased pathogenicity to *Pisum sativum* by carrying the *PDA1* gene, which provides resistance to pisatin, an antimicrobial compound produced by the host (Han et al. 2001). Interestingly, our genomic analysis revealed the presence of 2 putative pisatin demethylase genes on the AC Chr 16 of *D. sapinea*, suggesting that they could be involved in defense against phytoalexins produced by the host. Similarly, ACs in *Fusarium oxysporum* have been implicated in overcoming host defenses post host penetration (Ma et al. 2010), 1 of 2 ACs in *Colletotrichum higginsianum* is directly associated with pathogenicity to *Arabidopsis thaliana* (Plaumann et al. 2018). In contrast to the abovementioned studies, our study found no direct correlation between the presence of ACs in *D. sapinea* and pathogenicity. Furthermore, the differences in isolate pathogenicity despite the presence or absence of ACs suggest that other genetic mechanisms unrelated to the ACs may be responsible for the observed differences in disease development. It is possible, however, that the ACs only produce a discernible effect under particular environmental conditions, host conditions, or host species (as discussed in Bertazzoni et al. 2018), which were not met in the pathogenicity trial of this study. Alternatively, these ACs may not contribute directly toward pathogenicity but play a role in the responses of *D. sapinea* to other biotic or abiotic factors.

It is possible that the ACs discovered in this study play a role in the ability of *D. sapinea* to colonize its host endophytically. In *Stagonosporopsis rhizophila*, for example, Wei et al. (2024) showed that the loss of an AC changed the phenotype of this fungus from being pathogenic to endophytic. *D. sapinea* is known to colonize its host without causing symptoms and to exist as a latent pathogen (Bihon et al. 2011). Given the common nature of this characteristic in *D. sapinea*, AC Chr 15 would be the most likely candidate to be involved in the process because it is present in most isolates. The AC Chr 15 also has a higher proportion of effectors compared to both the CCs and Chr 16. Effectors, as a class of proteins, are known to play a role in evading host immunity or manipulating host responses (Li et al. 2024).

The 2 ACs found in isolates of *D. sapinea* were not distributed equally among the isolates investigated. Chr 15 occurs at a high frequency across multiple populations that span 2 countries in Africa, 9 in Europe, 2 in Asia, 3 in South America, 2 North American countries, and 1 in Oceania. In contrast, Chr 16 was found only in a single isolate of *D. sapinea* from Sweden. One explanation for these different patterns of association is that the acquisition of Chr 16 may have been a recent event, and as a result, this AC has not become established within all *D. sapinea* populations. Alternatively, this AC may only provide an advantage under particular conditions (Bertazzoni et al. 2018) and is only maintained at high frequency in certain populations of *D. sapinea*.

The provision of a chromosome-level assembly for *D. sapinea* in this study represents a valuable resource providing for new avenues of investigation on this pathogen in general and contributes to its potential for the development of an experimental system for this latent tree pathogen. For example, Oostlander et al. (2023, 2024) developed sporulation, infection, and transformation tools for the study of infection and host–pathogen interaction that can now be exploited more precisely to study gene function in this process. In addition, the discovery of ACs in *D. sapinea* provides intriguing new opportunities to gain insights into the biology and evolution of this pathogen through reverse genetics or through the curing of isolates from one or more of these ACs. Collectively, these new avenues of study should answer the many questions relating to the latent and pathogenic lifestyles of *D. sapinea*.

## Supplementary Material

jkaf239_Supplementary_Data

## Data Availability

The genome assemblies of 3 *D. sapinea* strains have been deposited in the NCBI GenBank database under bioproject number PRJNA1240814. The short- and long-read sequence data have been deposited in the NCBI Sequence Read Archive (SRA), and the accession numbers are provided in Supplementary Table 1. The pipelines and scripts used for data analyses are available on GitHub: https://github.com/PLockeS/Dsapinea_accessory_chromosomes. Genome assemblies, annotations, and variant call file have been deposited in FigShare DOI: 10.6084/m9.figshare.30271870. Supplemental material available at G3 online.
