# Supplementary material for: Chromosome-level genome assemblies for the latent pine pathogen, Diplodia sapinea, reveal two accessory chromosomes with distinct genomic features and evolutionary dynamics
Source: G3 (Bethesda). 2025 Oct 7;15(12):jkaf239. doi: 10.1093/g3journal/jkaf239 (PMC12693491; doi:10.1093/g3journal/jkaf239)
Supplement: jkaf239_Supplementary_Data [file jkaf239_supplementary_data.docx]

**Supplementary material**

**Supplementary Table 1.** Isolates of *D. sapinea* used for genome sequencing and analyses.

| **Isolate** | **Host** | **Country of isolation** | **Illumina accession** | **Nanopore accession** |
| --- | --- | --- | --- | --- |
| CMW39103 | *Pinus patula* | South Africa | [SRR32830896](https://dataview.ncbi.nlm.nih.gov/object/SRR32830896) | [SRR32830895](https://dataview.ncbi.nlm.nih.gov/object/SRR32830895) |
| CMW190 | *Pinus banksiana* | South Africa | [SRR32830892](https://dataview.ncbi.nlm.nih.gov/object/SRR32830892) | [SRR32830891](https://dataview.ncbi.nlm.nih.gov/object/SRR32830891) |
| CMW45410 | *Pinus nigra* | Sweden | [SRR32830890](https://dataview.ncbi.nlm.nih.gov/object/SRR32830890) | [SRR32830889](https://dataview.ncbi.nlm.nih.gov/object/SRR32830889) |
| CBS109727 | *Pinus radiata* | South Africa | [SRR32830888](https://dataview.ncbi.nlm.nih.gov/object/SRR32830888) | N/A |
| CBS119938 | *Pinus radiata* | Italy | [SRR32830887](https://dataview.ncbi.nlm.nih.gov/object/SRR32830887) | N/A |
| CBS120833 | *Prunus persica* | South Africa | [SRR32830886](https://dataview.ncbi.nlm.nih.gov/object/SRR32830886) | N/A |
| CBS623.74 | *Pinus radiata* | Chile | [SRR32830885](https://dataview.ncbi.nlm.nih.gov/object/SRR32830885) | N/A |
| 1684n^e^6-1 | *Pinus pinaster* | France | [SRR32830894](https://dataview.ncbi.nlm.nih.gov/object/SRR32830894) | N/A |
| Pier4 | *Pinus nigra* | France | [SRR32830893](https://dataview.ncbi.nlm.nih.gov/object/SRR32830893) | N/A |

**Supplementary Table 2.** Species of *Botryosphaeriaceae* used in protein clustering and phylogenomic analysis.

| **Genus** | **Species** | **Isolate** | **Genome accession** | **Original source** | **References** |
| --- | --- | --- | --- | --- | --- |
| ***Botryosphaeria*** | *dothidea* | CMW8000 | N/A | (Marsberg, et al. 2017) | (Nagel, et al. 2021) |
|  | *dothidea* | sdau11-99 | WWBZ00000000 | (Yu, et al. 2021) | (Yu, et al. 2021) |
|  | *kuwatsukai* | LW030101 | MDSR00000000 | (Liu, et al. 2016) | (Nagel, et al. 2021) |
| ***Diplodia*** | *corticola* | CBS112549 | MNUE01000001 | (Fernandes, et al. 2014) | (Fernandes, et al. 2014) |
|  | *scrobiculata* | CMW30223 | LAEG00000000 | (Wingfield, et al. 2015) | (Nagel, et al. 2021) |
|  | *seriata* | F98.1 | MSZU00000000 | (Robert-Siegwald, et al. 2017) | (Nagel, et al. 2021) |
|  | *seriata* | DS831 | LAQI00000000 | (Morales-Cruz, et al. 2015) | (Nagel, et al. 2021) |
| ***Eutiarosporella*** | *darliae* | 2G6 | GFXH01000000 | (Thynne, et al. 2019) | (Nagel, et al. 2021) |
|  | *pseudodarliae* | V4B6 | GFXI01000000 | (Thynne, et al. 2019) | (Nagel, et al. 2021) |
|  | *tritici-australis* | 153 | GFXG01000000 | (Thynne, et al. 2019) | (Nagel, et al. 2021) |
| ***Lasiodiplodia*** | *gonubiensis* | CBS115812 | RHKH00000000 | (Nagel, et al. 2021) | (Nagel, et al. 2021) |
|  | *pseudotheobromae* | CBS116459 | RHKG00000000 | (Nagel, et al. 2021) | (Nagel, et al. 2021) |
|  | *theobromae* | CBS164.96 | RHKF00000000 | (Nagel, et al. 2021) | (Nagel, et al. 2021) |
|  | *theobromae* | AM2As | QCYV00000000 | (Ali, et al. 2020) | (Ali, et al. 2020) |
|  | *theobromae* | CSS-01s | MDYX00000000 | (Yan, et al. 2018) | (Yan, et al. 2018) |
|  | *theobromae* | LA-SOL3 | VCHE00000000 | (Félix, et al. 2019) | (Félix, et al. 2019) |
| ***Macrophomina*** | *phaseolina* | MS6 | AHHD00000000 | (Islam, et al. 2012) | (Nagel, et al. 2021) |
| ***Neofusicoccum*** | *cordaticola* | CBS123634 | RHKC00000000 | (Nagel, et al. 2021) | (Nagel, et al. 2021) |
|  | *cordaticola* | CBS123638 | RHKD00000000 | (Nagel, et al. 2021) | (Nagel, et al. 2021) |
|  | *kwambonambiense* | CBS123639 | RHKE00000000 | (Nagel, et al. 2021) | (Nagel, et al. 2021) |
|  | *kwambonambiense* | CBS123642 | RKSS00000000 | (Nagel, et al. 2021) | (Nagel, et al. 2021) |
|  | *parvum* | CBS123649 | RHJY00000000 | (Nagel, et al. 2021) | (Nagel, et al. 2021) |
|  | *parvum* | CMW9080 | RHJX00000000 | (Nagel, et al. 2021) | (Nagel, et al. 2021) |
|  | *parvum* | UCRNP2 | AORE00000000 | (Blanco-Ulate, et al. 2013) | (Nagel, et al. 2021) |
|  | *ribis* | CBS121.26 | RHKA00000000 | (Nagel, et al. 2021) | (Nagel, et al. 2021) |
|  | *ribis* | CBS115475 | RHJZ00000000 | (Nagel, et al. 2021) | (Nagel, et al. 2021) |
|  | *umdonicola* | CBS123644 | RHKB00000000 | (Nagel, et al. 2021) | (Nagel, et al. 2021) |

**Supplementary Table 3.** Primer design for detection of ACs in *D. sapinea*.

| **Chr** | **Primer name** | **Primer seq (5’→3’)** | **Tm (℃)** | **Amplicon (bp)** | **Targeted gene** | **Targeted gene function** |
| --- | --- | --- | --- | --- | --- | --- |
| Control | DSBt_F | CTGCCTTCTGGTTTGTTGCC | 60.0 | 804 | g10611.t1 | Beta-tubulin |
|  | DSBt_R | TGCAGATGTCGTACAGAGCC | 59.8 |  |  |  |
| Chr 15 | Ch15_1F | GGCATCCAGGGACTTCGATT | 59.8 | 210 | g7929.t1 | Hypothetical protein |
|  | Ch15_1R | AGGGAGAGAGAGCTGTTGGT | 59.9 |  |  |  |
|  | Ch15_2F | CAATACCCCTCCGTTGTCGT | 59.8 | 415 | g7939.t1 | PKS |
|  | Ch15_2R | CGTGCGAGATGGTAGAGGAC | 60.0 |  |  |  |
|  | Ch15_3F | CGGTTTCGGTCTCCTGGAAA | 60.0 | 640 | g7955.t1 | Toxin synthesis |
|  | Ch15_3R | GATGATCTCGTCCCGGTAGG | 59.1 |  |  |  |
| Chr 16 | Ch16_1F | CCTCCGTTAGCCTTTGTCGT | 60.0 | 186 | CMW45410_12287-RA | C6 finger domain protein |
|  | Ch16_1R | CCACACACCCGACACTTGTA | 59.9 |  |  |  |
|  | Ch16_2F | TGCTAGCCCACCTGGATTTG | 60.0 | 419 | CMW45410_g2720.t1 | Hypothetical protein |
|  | Ch16_2R | GACGGCGTAGAGTTCCTAGC | 60.0 |  |  |  |
|  | Ch16_3F | GGCGGATATGAATGTCGGGT | 60.0 | 626 | CMW45410_12231-RA | NACHT and WD40 domain |
|  | Ch16_3R | CATCGAGAAGCTTGCGTTCG | 60.0 |  |  |  |

**Supplementary Table 4.** Results of PCR assays to identify dispensable chromosomes in *D. sapinea* isolates from various countries around the world.

| **Isolate (CMW)** | **Chr 15** | **Chr 16** | **Country** |
| --- | --- | --- | --- |
| 39103 | - | - | South Africa |
| 190 | + | - | South Africa |
| 45410 | + | + | Sweden |
| 29644 | + | - | Ethiopia |
| 4237 | + | - | South Africa |
| 29133 | + | - | South Africa |
| 29309 | + | - | South Africa |
| 29311 | + | - | South Africa |
| 31572 | + | - | South Africa |
| 31649 | + | - | South Africa |
| 31655 | + | - | South Africa |
| 32008 | + | - | South Africa |
| 34302 | + | - | South Africa |
| 24705 | + | - | China |
| 24707 | + | - | China |
| 24708 | + | - | China |
| 4889 | - | - | Indonesia |
| 32287 | - | - | Indonesia |
| 32361 | - | - | Indonesia |
| 34220 | - | - | Indonesia |
| 8750 | + | - | Great Britain |
| 33544 | + | - | Great Britain |
| 39331 | + | - | Montenegro* |
| 39333 | + | - | Montenegro |
| 39337 | + | - | Montenegro |
| 39341 | + | - | Montenegro |
| 8749 | + | - | Netherlands |
| 33543 | + | - | Netherlands |
| 39329 | + | - | Serbia |
| 39338 | + | - | Serbia |
| 39342 | + | - | Serbia |
| 32277 | + | - | Switzerland* |
| 8754 | + | - | USA |
| 31975 | + | - | Brazil |
| 14971 | + | - | Chile |
| 14974 | + | - | Chile |
| 5850 | + | - | Colombia |
| 32112 | + | - | Colombia |
| 32425 | + | - | New Zealand |
| 32431 | + | - | New Zealand |

**Supplementary Table 5.** Summary of *D. sapinea* isolates used in pathogenicity trials.

| **Isolate (CMW)** | **Origin** | **Host** | **DCs** |
| --- | --- | --- | --- |
| 39103 | South Africa | *P. patula* | - |
| 4889 | Indonesia | *P. patula* | - |
| 34220 | Indonesia | *P. patula* | - |
| 29644 | Ethiopia | *P. patula* | Chr 15 |
| 8754 | USA | *Pseudotsuga menziessi* | Chr 15 |
| 45410 | Sweden | *P. nigra* | Chr 15, Chr 16 |

**Supplementary Table 6.** Statistics of genome assemblies produced by Canu and Flye for each *D. sapinea* isolate.

| **Isolate** | **Assembler** | **Contigs** | **Size (Mb)** | **N50** | **L50** | **Mis-joins** | **Splits** |
| --- | --- | --- | --- | --- | --- | --- | --- |
| CMW39103 | Canu | 14 | 36.88 | 2859033 | 6 | 0 | 0 |
|  | Flye | 13 | 36.7 | 2852626 | 6 | 1 | 0 |
| CMW190 | Canu | 16 | 37.98 | 2831583 | 6 | 0 | 1 |
|  | Flye | 13 | 37.27 | 3188804 | 5 | 2 | 0 |
| CMW45410 | Canu | 18 | 38.07 | 2676158 | 7 | 0 | 2 |
|  | Flye | 15 | 37.93 | 2861173 | 5 | 1 | 0 |

**Supplementary Table 7.** Homologous genes within and between the accessory chromosomes

| **Orthogroup** | **Gene ID** | **Functions** | **Gene coordinates** |
| --- | --- | --- | --- |
| OG0000046 | CMW45410_12209-RA | Hypothetical protein | C16: 315435–317112 |
|  | CMW45410_g2750.t1 | Phosphatidylserine decarboxylase | C16: 454129–455696 |
|  | CMW45410_g7366.t1 | Hypothetical protein | C15: 67836–69319 |
| OG0000109 | CMW45410_g2702.t1 | Uracil phosphoribosyltransferase | C16 :94575–95528 |
|  | CMW45410_g2703.t1 | Uracil phosphoribosyltransferase | C16: 95772–96557 |
|  | CMW45410_g7406.t1 | Uracil phosphoribosyltransferase | C15: 372465–374576 |
| OG0000244 | CMW45410_g2740.t1 | TPR-like protein | C16: 376859–379465 |
|  | CMW45410_g2742.t1 | Kinesin light chain | C16: 383637–387395 |
| OG0001324 | CMW45410_g2714.t1 | p-loop containing nucleoside triphosphate hydrolase | C16: 168319–177832 |
|  | CMW45410_g2744.t1 | p-loop containing nucleoside triphosphate hydrolase | C16: 394379–401270 |
| OG0007116 | CMW45410_g2730.t2 | Pisatin demethylase | C16: 278952–280650 |
|  | CMW45410_g2754.t2 | Pisatin demethylase | C16: 473573–475271 |
| OG0008997 | CMW45410_12211-RA | Intracellular protein transport protein | C16: 322762–324416 |
|  | CMW45410_g2721.t1 | Hypothetical protein | C16: 221616–222379 |
| OG0012891 | CMW45410_12275-RA | P-loop containing nucleoside triphosphate hydrolase protein | C16: 563756–565406 |
|  | CMW45410_g2766.t1 | MMR-HSR1 multi-domain protein | C16: 566501–567674 |
| OG0013704 | CMW45410_g2719.t1 | FabD/lysophospholipase-like protein | C16: 214039–217469 |
|  | CMW45410_g2761.t1 | FabD/lysophospholipase-like protein | C16: 522608–525852 |
|  | CMW45410_12323-RA | FabD/lysophospholipase-like protein | C15: 117129–118392 |
| OG0013710 | CMW45410_12130-RA | Hypothetical protein | C16: 11300–12803 |
|  | CMW45410_12193-RA | Hypothetical protein | C16: 254769–255704 |
|  | CMW45410_12258-RA | Hypothetical protein | C16: 500049–501552 |
|  | CMW45410_12329-RA | Hypothetical protein | C15: 131632–132168 |
| OG0014162 | CMW45410_12215-RA | Transcription factor jumonji/aspartyl beta-hydroxylase | C16: 330645–333686 |
|  | CMW45410_12241-RA | Transcription factor jumonji/aspartyl beta-hydroxylase | C16: 443975–446881 |
|  | CMW45410_12305-RA | Transcription factor jumonji/aspartyl beta-hydroxylase | C15: 59333–63116 |
| OG0014167 | CMW45410_g2691.t1 | DUF221-domain-containing protein | C16: 1185–4726 |
|  | CMW45410_g2716.t1 | DUF221-domain-containing protein | C16: 199774–202146 |
|  | CMW45410_g2717.t1 | Hypothetical protein | C16: 202303–203157 |
|  | CMW45410_g2759.t1 | DUF221-domain-containing protein | C16: 508290–511664 |
|  | CMW45410_12319-RA | Phosphate metabolism protein | C15: 95453–97867 |
| OG0014168 | CMW45410_g2718.t1 | G-protein coupled receptor | C16: 206172–207859 |
|  | CMW45410_g2726.t1 | G-protein coupled receptor | C16: 250343–251956 |
|  | CMW45410_g2760.t1 | G-protein coupled receptor | C16: 514744–516431 |
| OG0014169 | CMW45410_12190-RA | Zinc finger CCHC-type protein | C16: 241789–242812 |
|  | CMW45410_g2725.t1 | Zinc finger CCHC-type protein | C16: 240811–241647 |
|  | CMW45410_g2746.t1 | Zinc finger CCHC-type protein | C16: 423511–425019 |
|  | CMW45410_g2769.t1 | Zinc finger CCHC-type protein | C16: 601214–602734 |
| OG0014834 | CMW45410_12205-RA | Hypothetical protein | C16: 303765–305447 |
|  | CMW45410_12206-RA | Putative aspartic-type endopeptidase | C16: 306016–307641 |
|  | CMW45410_12330-RA | Serine/threonine-protein kinase PKH1 | C15: 133670–135262 |
| OG0014978 | CMW45410_g2692.t1 | Hypothetical protein | C16: 12943–13647 |
|  | CMW45410_g2727.t1 | Hypothetical protein | C16: 256362–257069 |
|  | CMW45410_g2758.t1 | Hypothetical protein | C16: 499205–499909 |
| OG0014979 | CMW45410_g2693.t1 | Hypothetical protein | C16: 28062–29127 |
|  | CMW45410_g2729.t1 | Hypothetical protein | C16: 272906–273821 |
|  | CMW45410_g2757.t1 | Hypothetical protein | C16: 483037–483932 |
| OG0014980 | CMW45410_g2731.t1 | Hypothetical protein | C16: 281650–282736 |
|  | CMW45410_g2755.t1 | Hypothetical protein | C16: 476271–477432 |
| OG0014981 | CMW45410_12137-RA | Transmembrane protein | C16: 41900–42374 |
|  | CMW45410_12281-RA | Transmembrane protein | C16: 590071–590545 |
| OG0014982 | CMW45410_12132-RA | Hypothetical protein | C16: 18990–19976 |
|  | CMW45410_12195-RA | F-box domain-containing protein | C16: 262746–263876 |
|  | CMW45410_12256-RA | Hypothetical protein | C16: 492018–493004 |
| OG0016363 | CMW45410_g2695.t1 | Hypothetical protein | C16: 46523–46834 |
|  | CMW45410_g2753.t1 | Hypothetical protein | C16: 469715–470569 |
|  | CMW45410_g2768.t1 | Hypothetical protein | C16: 594450–595004 |
| OG0016364 | CMW45410_g2722.t1 | N/A | C16: 232339–233148 |
|  | CMW45410_g2762.t1 | N/A | C16: 528326–529234 |
| OG0016365 | CMW45410_g2738.t1 | Rhodopsin kinase | C16: 362628–365091 |
|  | CMW45410_g2765.t1 | Rhodopsin kinase | C16: 554467–556930 |
| OG0016367 | CMW45410_12133-RA | Hypothetical protein | C16: 21051–21999 |
|  | CMW45410_12255-RA | Hypothetical protein | C16: 490090–490944 |
| OG0016368 | CMW45410_12141-RA | Hypothetical protein | C16: 53766–56390 |
|  | CMW45410_12207-RA | Hypothetical protein | C16: 311894–313462 |
|  | CMW45410_12208-RA | Hypothetical protein | C16: 313755–314756 |
| OG0016369 | CMW45410_12134-RA | Hypothetical protein | C16: 25729–26427 |
|  | CMW45410_12197-RA | Hypothetical protein | C16: 270021–271837 |
|  | CMW45410_12254-RA | Hypothetical protein | C16: 485567–486361 |
| OG0016371 | CMW45410_12185-RA | Ferric reductase | C16: 228420–230259 |
|  | CMW45410_12188-RA | Putative cell surface metalloreductase | C16: 235049–236748 |
|  | CMW45410_12268-RA | Ferric reductase | C16: 531178–533015 |
| OG0017888 | CMW45410_12129-RA | Proteophosphoglycan | C16: 9987–10744 |
|  | CMW45410_12259-RA | Proteophosphoglycan | C16: 502108–502865 |
| OG0017889 | CMW45410_12128-RA | Hypothetical protein | C16: 5765–7402 |
|  | CMW45410_12260-RA | Hypothetical protein | C16: 505448–507084 |
| OG0017891 | CMW45410_12146-RA | Hypothetical protein | C16: 66569–67644 |
|  | CMW45410_12278-RA | Hypothetical protein | C16: 578974–580049 |
| OG0017892 | CMW45410_12145-RA | Start control protein | C16: 63841–64439 |
|  | CMW45410_12279-RA | Start control protein | C16: 582177–582775 |
| OG0000215 | CMW45410_g2701.t1 | GTP cyclohydrolase I | C16: 92482–93352 |
|  | CMW45410_g7405.t1 | GTP cyclohydrolase I | C15: 370359–371229 |
| OG0000243 | CMW45410_g2706.t1 | NFX1-type zinc finger-containing protein 1 | C16: 133492 139435 |
|  | CMW45410_g7410.t1 | NFX1-type zinc finger-containing protein 1 | C15: 404797–410735 |
| OG0010181 | CMW45410_12161-RA | Hypothetical protein | C16: 123447–125303 |
|  | CMW45410_g7409.t1 | Hypothetical protein | C15: 395743–396060 |
| OG0011053 | CMW45410_g2700.t1 | Phosphoribosyl transferase domain protein | C16: 87980–91444 |
|  | CMW45410_g7404.t1 | Phosphoribosyl transferase domain protein | C15: 365853–369317 |
| OG0012849 | CMW45410_g2748.t1 | Hypothetical protein | C16: 433120–434727 |
|  | CMW45410_g7364.t1 | Hypothetical protein | C15: 48790–49929 |
| OG0012895 | CMW45410_12238-RA | Hypothetical protein | C16: 435115–436415 |
|  | CMW45410_12302-RA | Hypothetical protein | C15: 50305–50964 |
| OG0014161 | CMW45410_g2751.t1 | Proline-rich receptor-like protein kinase perk4-like | C16: 456861–458762 |
|  | CMW45410_g7367.t1 | Ring-infected erythrocyte surface antigen-like | C15: 71062–72243 |
| OG0014968 | CMW45410_g2694.t1 | Putative serine threonine protein kinase | C16: 35405–37855 |
|  | CMW45410_g7396.t1 | Serine threonine protein kinase | C15: 288856–291087 |
| OG0016340 | CMW45410_12154-RA | Ribonuclease III | C16: 98211–100232 |
|  | CMW45410_12381-RA | Ribonuclease III | C15: 376050–377996 |
| OG0016345 | CMW45410_g2749.t1 | Hypothetical protein | C16: 450269–450889 |
|  | CMW45410_g7365.t1 | Hypothetical protein | C15: 66495–67115 |
| OG0016347 | CMW45410_12156-RA | Ubiquinol-cytochrome-c reductase cytochrome c1 | C16: 111204–111733 |
|  | CMW45410_12383-RA | Ubiquinol-cytochrome-c reductase cytochrome c1 | C15: 385869–386276 |
| OG0016348 | CMW45410_12248-RA | Hypothetical protein | C16: 461733–463723 |
|  | CMW45410_12311-RA | Hypothetical protein | C15: 74938–76930 |
| OG0008436 | CMW45410_g7358.t1 | UPF0394 membrane protein | C15: 23570–23914 |
|  | CMW45410_g7359.t1 | UPF0394 membrane protein | C15: 23932–24593 |
| OG:0008659 | CMW45410_g7356.t1 | Fungal-specific transcription factor domain-containing protein | C15: 20930–21642 |
|  | CMW45410_g7357.t1 | Fungal specific transcription factor | C15: 21723–23417 |

**Supplementary Table 8.** Summary of all isolates used in the current study and whether the DCs are present or absent.

| **Isolate** | **Chr 15** | **Chr 16** | **Evidence** | **Country** | **Source** |
| --- | --- | --- | --- | --- | --- |
| CMW39103 | - | - | Whole genome alignment | South Africa | Current study |
| CMW190 | + | - | Whole genome alignment | South Africa | Current study |
| CMW45410 | + | + | Whole genome alignment | Sweden | Current study |
| CMW29644 | + | - | PCR assay | Ethiopia | Current study |
| CMW4237 | + | - | PCR assay | South Africa | Current study |
| CMW29133 | + | - | PCR assay | South Africa | Current study |
| CMW29309 | + | - | PCR assay | South Africa | Current study |
| CMW29311 | + | - | PCR assay | South Africa | Current study |
| CMW31572 | + | - | PCR assay | South Africa | Current study |
| CMW31649 | + | - | PCR assay | South Africa | Current study |
| CMW31655 | + | - | PCR assay | South Africa | Current study |
| CMW32008 | + | - | PCR assay | South Africa | Current study |
| CMW34302 | + | - | PCR assay | South Africa | Current study |
| CBS109727 | + | - | Illumina resequencing | South Africa | Current study |
| CBS120833 | + | - | Illumina resequencing | South Africa | Current study |
| ZXD319 | - | - | Whole genome alignment | China | (Wang et al. 2025) |
| KE8364 | + | - | Whole genome alignment | China | (Yu, et al. 2022) |
| KE8391 | + | - | Whole genome alignment | China | (Yu, et al. 2022) |
| KE8634 | + | - | Whole genome alignment | China | (Yu, et al. 2022) |
| CMW24705 | + | - | PCR assay | China | Current study |
| CMW24707 | + | - | PCR assay | China | Current study |
| CMW24708 | + | - | PCR assay | China | Current study |
| CMW4889 | - | - | PCR assay | Indonesia | Current study |
| CMW32287 | - | - | PCR assay | Indonesia | Current study |
| CMW32361 | - | - | PCR assay | Indonesia | Current study |
| CMW34220 | - | - | PCR assay | Indonesia | Current study |
| 162220 | - | - | Whole genome alignment | Singapore | GCA_964300245.1 |
| 162225 | - | - | Whole genome alignment | Singapore | GCA_964300275.1 |
| 142926 | + | - | Whole genome alignment | Estonia | GCA_964300335.1 |
| 1684n^e^6-1 | + | - | Illumina resequencing | France | Current study |
| Pier4 | + | - | Illumina resequencing | France | Current study |
| CMW8750 | + | - | PCR assay | Great Britain | Current study |
| CMW33544 | + | - | PCR assay | Great Britain | Current study |
| CBS119938 | + | - | Illumina resequencing | Italy | Current study |
| CMW39331 | + | - | PCR assay | Montenegro | Current study |
| CMW39333 | + | - | PCR assay | Montenegro | Current study |
| CMW39337 | + | - | PCR assay | Montenegro | Current study |
| CMW39341 | + | - | PCR assay | Montenegro | Current study |
| CMW8749 | + | - | PCR assay | Netherlands | Current study |
| CMW33543 | + | - | PCR assay | Netherlands | Current study |
| 150338 | + | - | Whole genome alignment | Russia | GCA_964300325.1 |
| 150346 | + | - | Whole genome alignment | Russia | GCA_964300295.1 |
| 163419 | + | - | Whole genome alignment | Russia | GCA_964300345.1 |
| 163102 | + | - | Whole genome alignment | Russia | GCA_964300265.1 |
| CMW39329 | + | - | PCR assay | Serbia | Current study |
| CMW39338 | + | - | PCR assay | Serbia | Current study |
| CMW39342 | + | - | PCR assay | Serbia | Current study |
| CMW32277 | + | - | PCR assay | Switzerland | Current study |
| 158147 | + | - | Whole genome alignment | Canada | GCA_964300305.1 |
| 157895 | + | - | Whole genome alignment | Canada | GCA_964300285.1 |
| CMW8754 | + | - | PCR assay | USA | Current study |
| CMW31975 | + | - | PCR assay | Brazil | Current study |
| 164087 | + | - | Whole genome alignment | Brazil | GCA_964300255.1 |
| 164117 | + | - | Whole genome alignment | Brazil | GCA_964300315.1 |
| CBS623.74 | + | - | Illumina resequencing | Chile | Current study |
| CMW14971 | + | - | PCR assay | Chile | Current study |
| CMW14974 | + | - | PCR assay | Chile | Current study |
| CMW5850 | + | - | PCR assay | Colombia | Current study |
| CMW32112 | + | - | PCR assay | Colombia | Current study |
| CMW32425 | + | - | PCR assay | New Zealand | Current study |
| CMW32431 | + | - | PCR assay | New Zealand | Current study |

**Supplementary Table 9.** Lesion lengths measured from pathogenicity trial.

| **Isolate** | **ACs** | **Lesion lengths (mm)** | **Average lesion length (mm)** |
| --- | --- | --- | --- |
| CMW39103 | None | 53  66  68  71  72  74  77 | 68.71 |
| CMW4889 | None | 7  13  57  65  78  88  97 | 57.86 |
| CMW34220 | None | 32  49  63  71  76  88  91 | 67.14 |
| CMW8754 | Chr 15 | 42  43  45  48  62  79  92 | 58.71 |
| CMW29644 | Chr 15 | 7  8  11  12  24  28  43 | 19 |
| CMW45410 | Chr 15, Chr 16 | 14  43  49  52  87  91  98 | 62 |


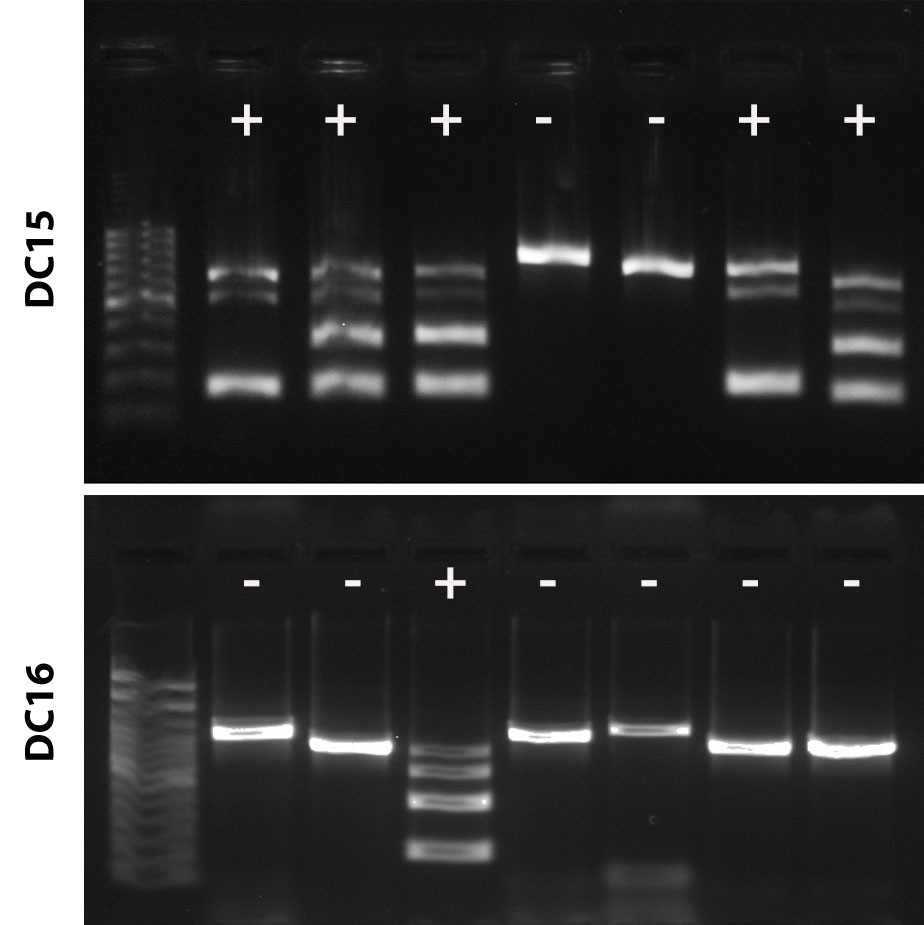


**Supplementary Figure 1.** Gel electrophoresis image showing multiplex PCR assays for the detection of each AC. A single amplicon shows samples without the AC, and multiple amplicons indicate the presence of the AC.


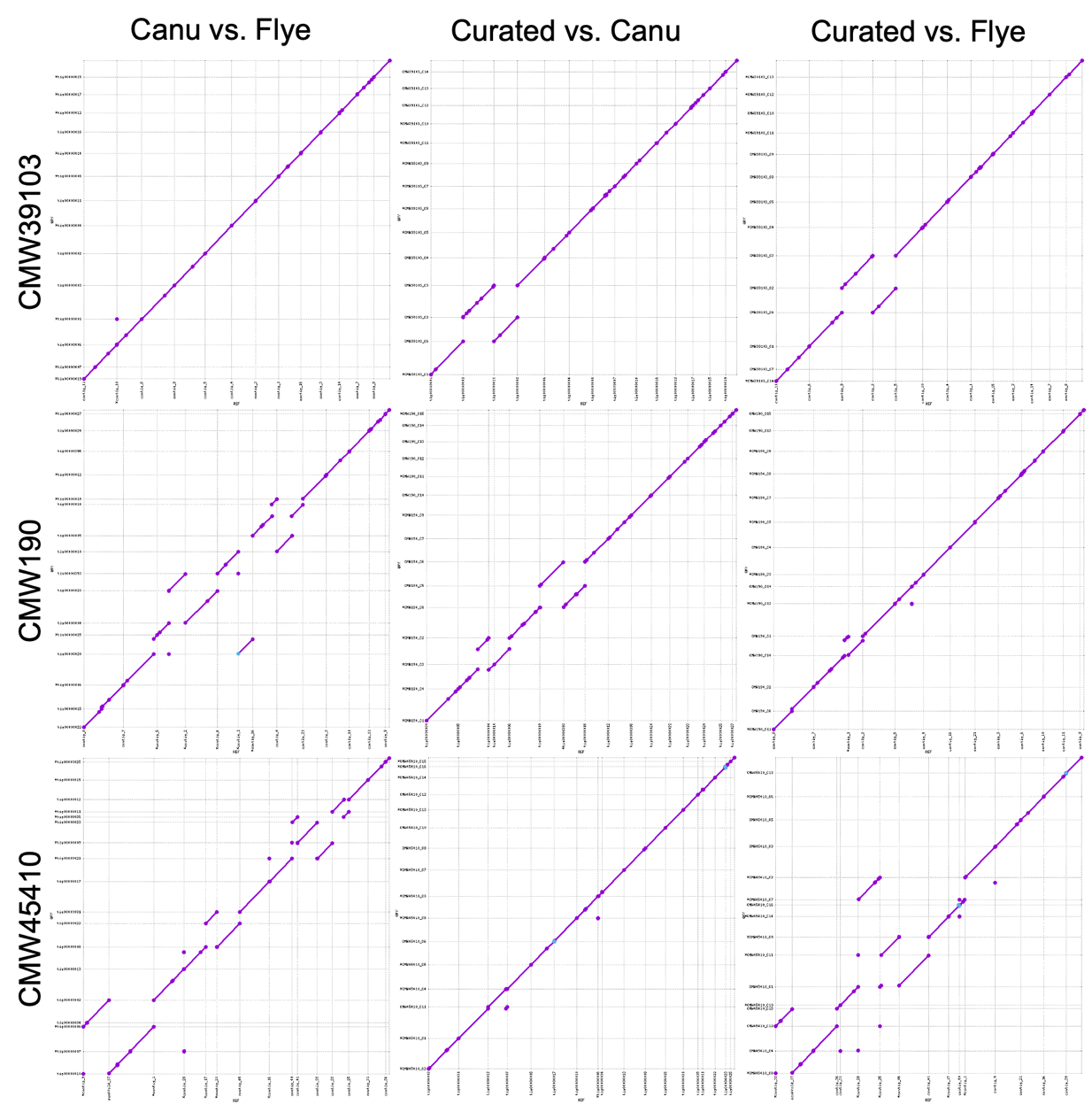


**Supplementary Figure 2.** Whole genome alignments generated using MUMmer of the initial assemblies and the final curated assemblies.


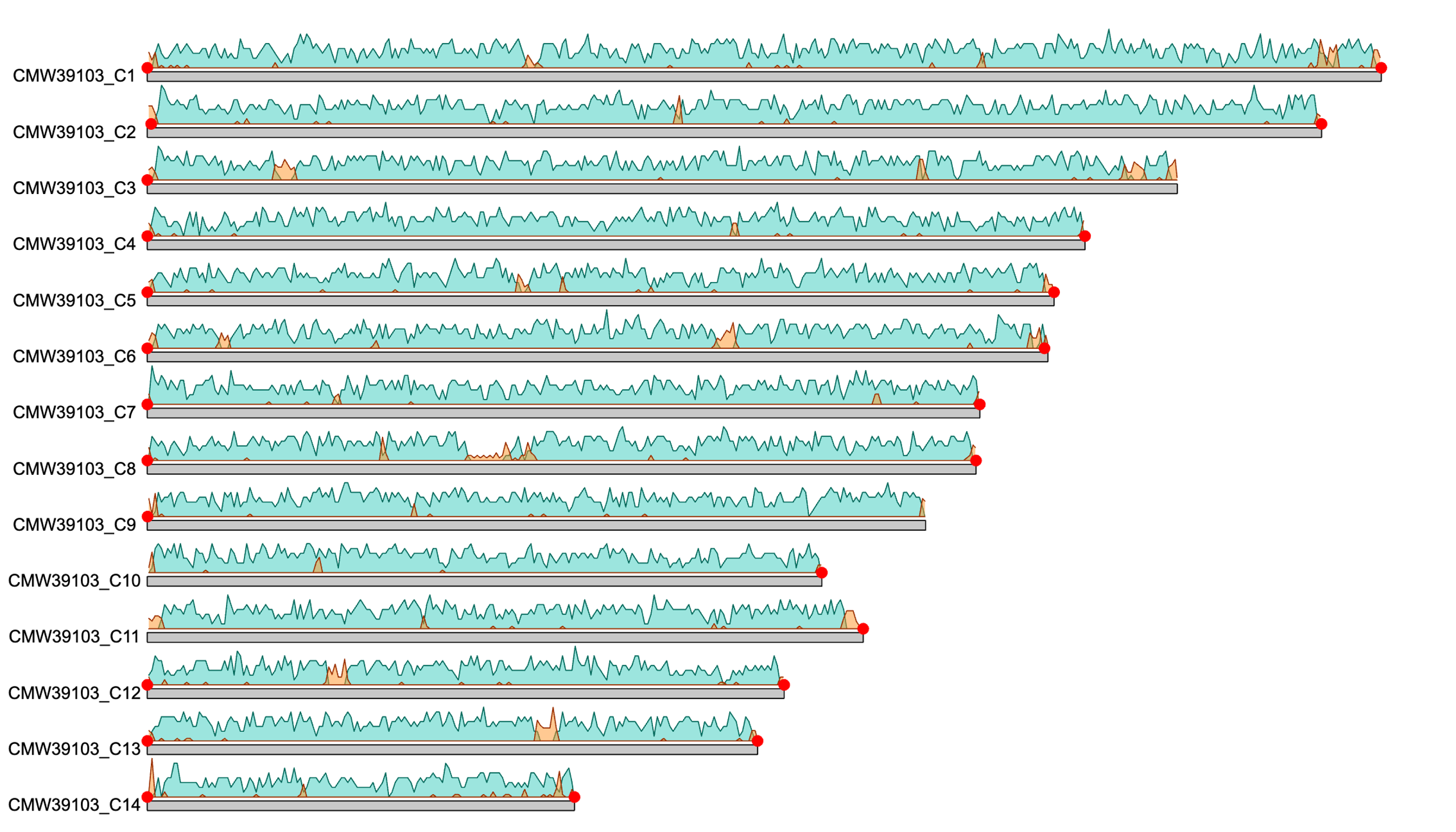


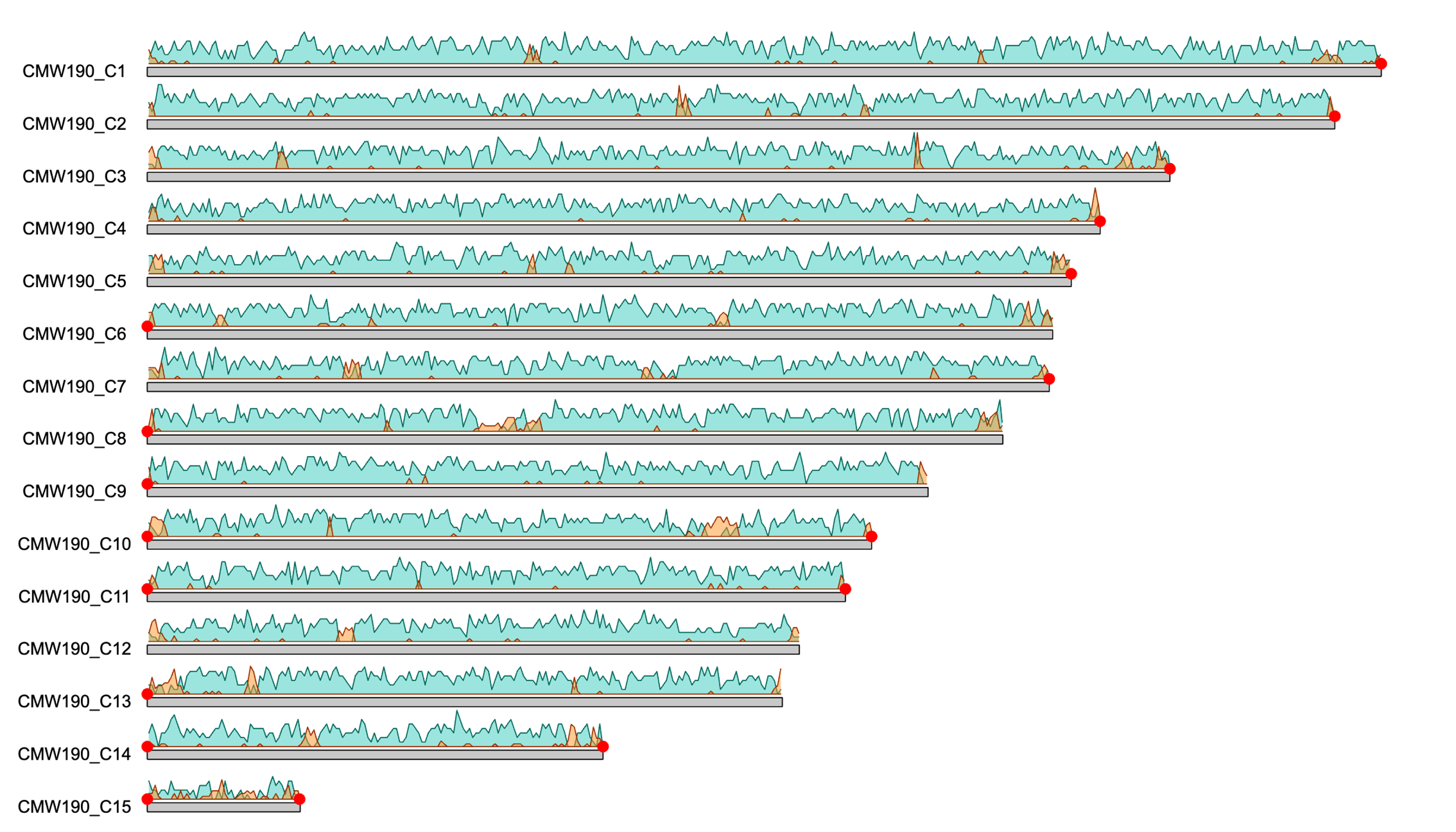


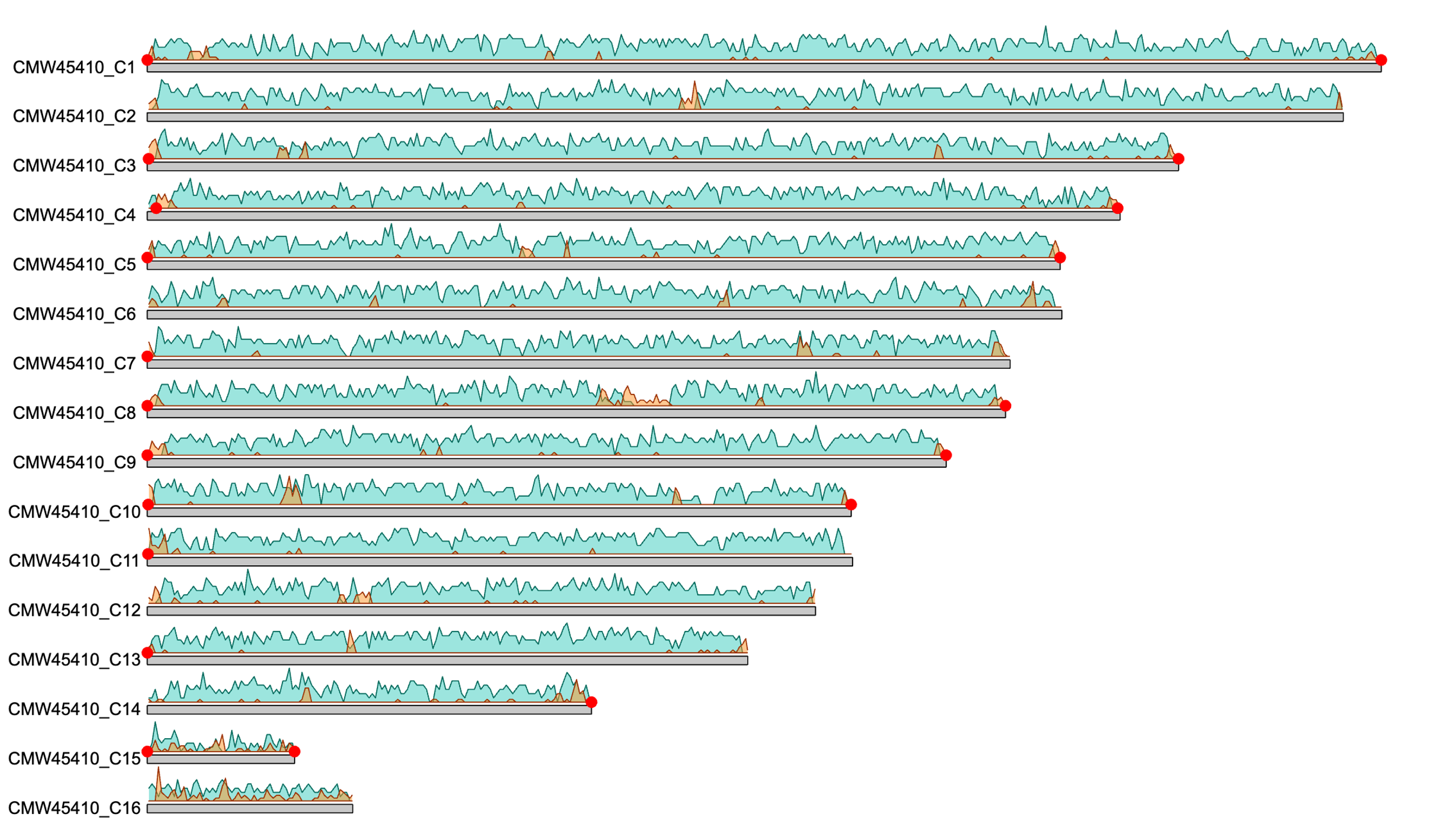


**Supplementary Figure 3.** Karyoplots of the genome assemblies generated in this study for CMW39103, CMW190 and CMW45410 using karyoploteR v1.28.0 using a window size of 10 Kb (Gel and Serra 2017). Grey bars represent the assembled contigs, blue density plots show gene density, and orange density plots show TE density. Red dots indicate an identified telomeric region.


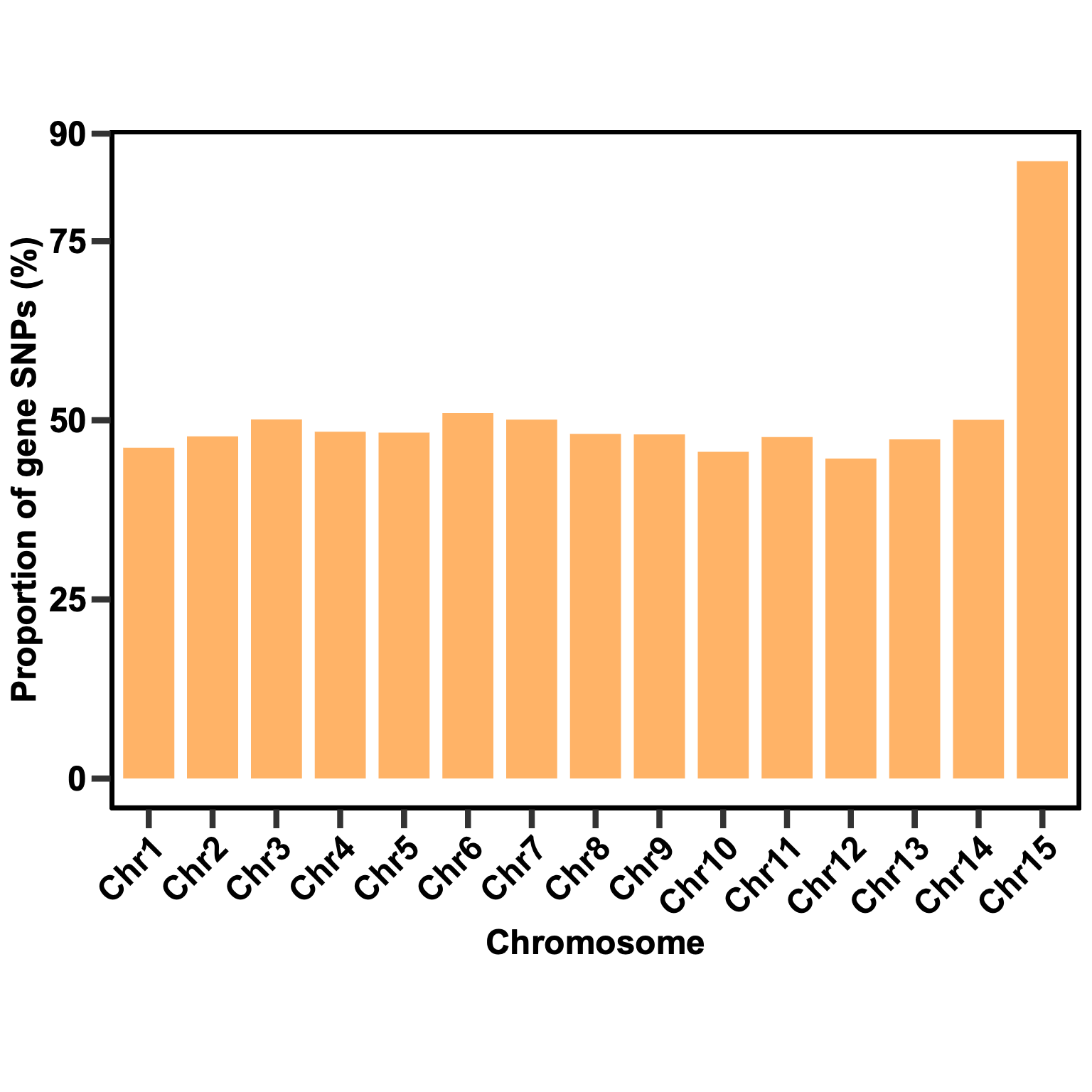


**Supplementary Figure 4.** Bar graph comparing the proportion of RIP-like SNPs in gene regions between each chromosome of CMW45410.


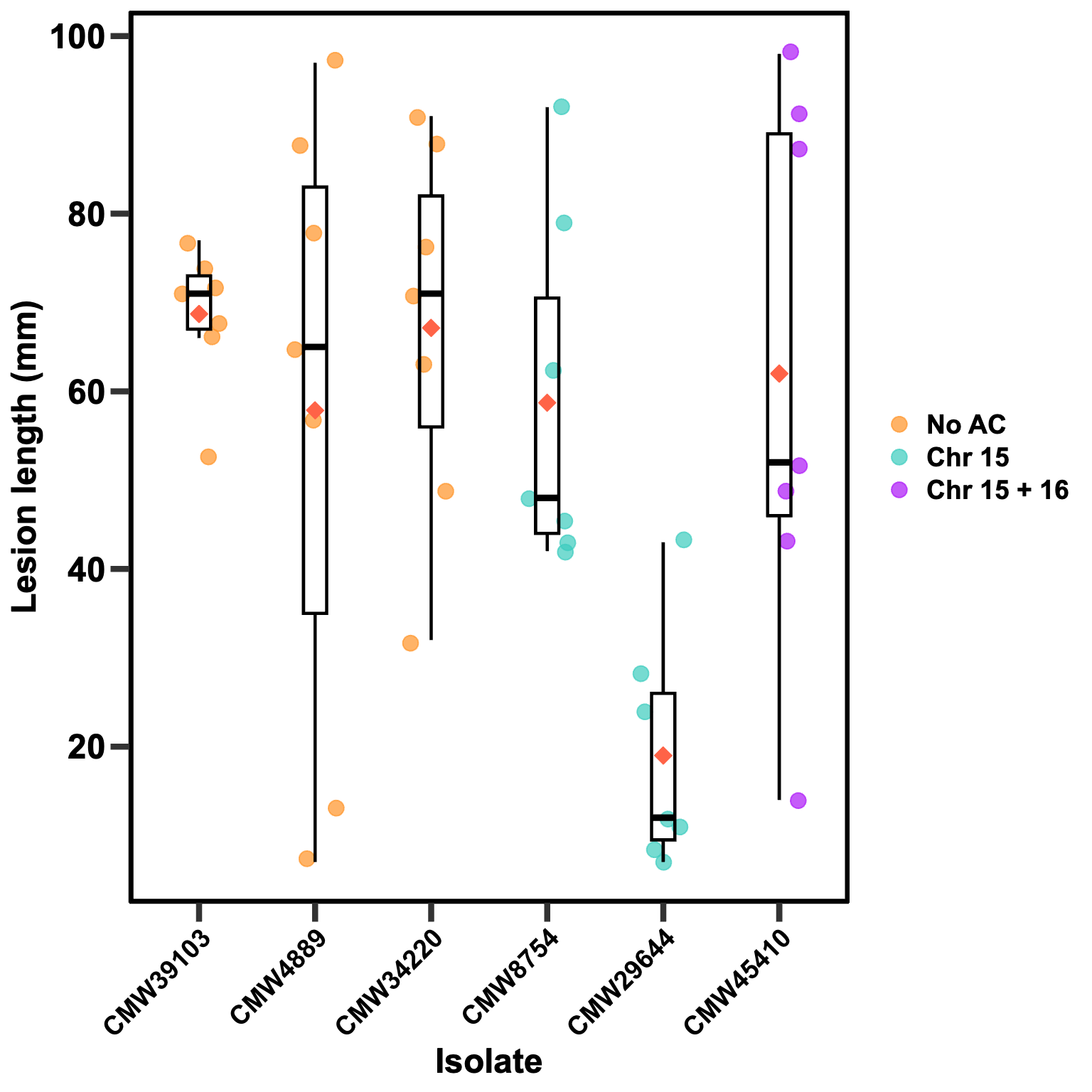


**Supplementary Figure 5.** Lesion measurements produced by *D. sapinea* isolates used in the pathogenicity trial.

**References**

Ali SS et al. 2020. Genome and transcriptome analysis of the latent pathogen *Lasiodiplodia theobromae*, an emerging threat to the cacao industry. Genome. 63(1):37–52. https://doi.org/10.1139/gen-2019-0112

Blanco-Ulate B, Rolshausen P, Cantu D. 2013. Draft genome sequence of *Neofusicoccum parvum* isolate UCR-NP2, a fungal vascular pathogen associated with grapevine cankers. Genome Announc. 1(3):e00339–00313. https://doi.org/10.1128/genomeA.00339-13.

Félix C et al. 2019. A multi-omics analysis of the grapevine pathogen *Lasiodiplodia theobromae* reveals that temperature affects the expression of virulence-and pathogenicity-related genes. Sci Repo. 9(1):1–12. https://doi.org/10.1038/s41598-019-49551-w.

Fernandes I, Alves A, Correia A, Devreese B, Esteves AC. 2014. Secretome analysis identifies potential virulence factors of *Diplodia corticola*, a fungal pathogen involved in cork oak (*Quercus suber*) decline. Fungal Biol. 118(5-6):516–523. https://doi.org/10.1016/j.funbio.2014.04.006.

Gel B, Serra E. 2017. KaryoploteR: an R/Bioconductor package to plot customizable genomes displaying arbitrary data. Bioinformatics. 33(19):3088–3090. https://doi.org/10.1093/bioinformatics/btx346.

Islam MS et al. 2012. Tools to kill: Genome of one of the most destructive plant pathogenic fungi *Macrophomina phaseolina*. BMC Genomics. 13(1):1–16. https://doi.org/10.1186/1471-2164-13-493.

Liu Z et al. 2016. Draft genome sequence of *Botryosphaeria dothidea*, the pathogen of apple ring rot. Genome Announc. 4(5):e01142–01116. https://doi.org/10.1128/genomeA.01142-16.

Marsberg A et al. 2017. *Botryosphaeria dothidea*: a latent pathogen of global importance to woody plant health. Mol Plant Pathol. 18(4):477–488. https://doi.org/10.1111/mpp.12495.

Morales-Cruz A et al. 2015. Distinctive expansion of gene families associated with plant cell wall degradation, secondary metabolism, and nutrient uptake in the genomes of grapevine trunk pathogens. BMC Genomics. 16(1):1–22. https://doi.org/10.1186/s12864-015-1624-z.

Nagel JH, Wingfield MJ, Slippers B. 2021. Increased abundance of secreted hydrolytic enzymes and secondary metabolite gene clusters define the genomes of latent plant pathogens in the *Botryosphaeriaceae*. BMC Genomics. 22(1):1–24. https://doi.org/10.1186/s12864-021-07902-w.

Robert-Siegwald G et al. 2017. Draft genome sequence of *Diplodia seriata* F98. 1, a fungal species involved in grapevine trunk diseases. Genome Announc. 5(14):e00061–00017. https://doi.org/10.1128/genomeA.00061-17.

Thynne E, Mead OL, Chooi Y-H, McDonald MC, Solomon PS. 2019. Acquisition and loss of secondary metabolites shaped the evolutionary path of three emerging phytopathogens of wheat. Genome Biol Evol. 11(3):890–905. https://doi.org/10.1093/gbe/evz037.

Wang Q, Liu F, Xu H, Zhou X. 2025. Whole-genome sequencing of global forest pathogen *Diplodia sapinea* causing pine shoot blight. BMC Genom Data. 26(1):1–4. https://doi.org/10.1186/s12863-025-01328-z.

Wingfield BD et al. 2015. Draft genome sequences of *Chrysoporthe austroafricana*, *Diplodia scrobiculata*, *Fusarium nygamai*, *Leptographium lundbergii*, *Limonomyces culmigenus*, *Stagonosporopsis tanaceti*, and *Thielaviopsis punctulata*. IMA Fungus. 6(1):233–248. https://doi.org/10.5598/imafungus.2015.06.01.15.

Yan JY et al. 2018. Comparative genome and transcriptome analyses reveal adaptations to opportunistic infections in woody plant degrading pathogens of *Botryosphaeriaceae*. DNA Res. 25(1):87-102. https://doi.org/10.1093/dnares/dsx040.

Yu C et al. 2021. Genome assembly and annotation of *Botryosphaeria dothidea* sdau11-99, a latent pathogen of apple fruit ring rot in China. Plant Dis. 105(05):1555-1557. https://doi.org/10.1094/PDIS-06-20-1182-A.

Yu C, Diao Y, Lu Q, Zhao J, Cui S, Xiong X, Lu A, Zhang X, Liu H. 2022. Comparative genomics reveals evolutionary traits, mating strategies, and pathogenicity-related genes variation of *Botryosphaeriaceae*. Front Microbiol. 13. https://doi.org/10.3389/fmicb.2022.800981.
